# Metabotropic glutamate receptor 5 inhibits α-synuclein-induced microglia inflammation to protect from neurotoxicity in Parkinson’s disease

**DOI:** 10.1186/s12974-021-02079-1

**Published:** 2021-01-18

**Authors:** Ya-Nan Zhang, Jing-Kai Fan, Li Gu, Hui-Min Yang, Shu-Qin Zhan, Hong Zhang

**Affiliations:** 1 Department of Neurobiology, School of Basic Medical Sciences, Beijing Key Laboratory of Neural Regeneration and Repair, Beijing Institute for Brain Disorders, Capital Medical University, Beijing, 100069 China; 2 Department of Neurology, Xuanwu Hospital, Capital Medical University, Beijing, 100053 China; 3 Beijing Key Laboratory of Neuromodulation, Beijing, 100053 China

**Keywords:** Metabotropic glutamate receptor 5, α-synuclein, Microglia, Inflammation, Parkinson’s disease

## Abstract

**Background:**

Microglia activation induced by α-synuclein (α-syn) is one of the most important factors in Parkinson’s disease (PD) pathogenesis. However, the molecular mechanisms by which α-syn exerts neuroinflammation and neurotoxicity remain largely elusive. Targeting metabotropic glutamate receptor 5 (mGluR5) has been an attractive strategy to mediate microglia activation for neuroprotection, which might be an essential regulator to modulate α-syn-induced neuroinflammation for the treatment of PD. Here, we showed that mGluR5 inhibited α-syn-induced microglia inflammation to protect from neurotoxicity in vitro and in vivo.

**Methods:**

Co-immunoprecipitation assays were utilized to detect the interaction between mGluR5 and α-syn in microglia. Griess, ELISA, real-time PCR, western blotting, and immunofluorescence assays were used to detect the regulation of α-syn-induced inflammatory signaling, cytokine secretion, and lysosome-dependent degradation.

**Results:**

α-syn selectively interacted with mGluR5 but not mGluR3, and α-syn N terminal deletion region was essential for binding to mGluR5 in co-transfected HEK293T cells. The interaction between these two proteins was further detected in BV2 microglia, which was inhibited by the mGluR5 specific agonist CHPG without effect by its selective antagonist MTEP. Moreover, in both BV2 cells and primary microglia, activation of mGluR5 by CHPG partially inhibited α-syn-induced inflammatory signaling and cytokine secretion and also inhibited the microglia activation to protect from neurotoxicity. We further found that α-syn overexpression decreased mGluR5 expression via a lysosomal pathway, as evidenced by the lysosomal inhibitor, NH_4_Cl, by blocking mGluR5 degradation, which was not evident with the proteasome inhibitor, MG132. Additionally, co-localization of mGluR5 with α-syn was detected in lysosomes as merging with its marker, LAMP-1. Consistently, in vivo experiments with LPS- or AAV-α-syn-induced rat PD model also confirmed that α-syn accelerated lysosome-dependent degradation of mGluR5 involving a complex, to regulate neuroinflammation. Importantly, the binding is strengthened with LPS or α-syn overexpression but alleviated by urate, a potential clinical biomarker for PD.

**Conclusions:**

These findings provided evidence for a novel mechanism by which the association of α-syn with mGluR5 was attributed to α-syn-induced microglia activation via modulation of mGluR5 degradation and its intracellular signaling. This may be a new molecular target for an effective therapeutic strategy for PD pathology.

**Supplementary Information:**

The online version contains supplementary material available at 10.1186/s12974-021-02079-1.

## Background

Parkinson’s disease (PD) is the second most common neuronal degenerative disorder, with selective degeneration of dopaminergic (DA) neurons in the substantia nigra pars compacta and the concomitant loss of nigrostriatal DAergic termini, along with the aggregation and accumulation of an intraneuronal protein complex called Lewy bodies, comprised of aggregated α-synuclein (α-syn) as the principal component. α-syn contains 140 amino acids, with five imperfect repeats with the consensus sequence, KTKEGV, in the N-terminal half; the non-amyloid-β (Aβ) component region, and an acidic C-terminal region. Overexpressed α-syn level due to the multiplication of SNCA alleles in PD patients plays a crucial role in the cause of PD [1, 2]. Extracellular α-syn stimulates abnormal microglia activation in PD models, accompanied by multiple intracellular signaling pathways involving nuclear factor (NF)-κB, phosphatidylinositol 3 kinase (PI3K)/protein kinase B (AKT), mitogen-activated protein kinases (MAPKs) [3–6], and a large number of inflammatory factors such as nitric oxide (NO), tumor necrosis factor (TNF)-α, interleukin (IL)-1β, and reactive oxygen species (ROS) secretions in vitro, which can lead to further microglia activation and cause a perpetuating cycle of neurotoxicity, aggravating neurodegenerative diseases [3, 7–10]. The microglia activation induced by α-syn is one of the most important factors in the pathogenesis of PD. However, the exact molecular mechanisms of how α-syn exerts neuroinflammation and neurotoxicity remain elusive.

Previous evidence demonstrated that α-syn can be secreted by neurons [11], engulfed, and degraded by neighboring cells, especially microglia and astrocytes, which contributes to the regulation of α-syn homeostasis in the brain [12]. Among the major brain cell types, microglia show the highest rate of degradation of α-syn aggregates in brain parenchyma [13]. Proteasomes and lysosomes likely play complementary roles in the removal of intracellular α-syn species, in a manner that depends on α-syn post-translational modifications. Given the observations that microglia activation can be induced by α-syn and is accompanied by α-syn aggregates degraded efficiently in microglia, it is important to identify the factors regulating this inflammatory process triggered by α-syn-induced microglia activation.

Metabotropic glutamate receptors (mGluRs) have stimulated widespread interest in neuroinflammation in recent years. These receptors are classified according to their receptor-coupled second messenger systems and specificity for different agonists into three groups; group I includes mGluR1/5, group II includes mGluR2/3, and group III includes mGluR4/6–8. mGluR5 is composed of an extracellular bilobate Venus flytrap (VFT) module, a heptahelical domain (HD), and a large intracellular C-terminal tail. The binding site for orthosteric ligands on mGluR5 such as (RS)-2-chloro-5-hydroxyphenylglycine (CHPG) is located in the VFT and the binding pocket of allosteric modulators such as 3-[(2-methyl-1, 3-thiazol-4-yl) ethynyl]-pyridine (MTEP), is located within the HD. The C-terminus contains several binding sites for intracellular proteins [14]. mGluR5 is closely related to the pathogenesis of various neurological disorders, including PD and Alzheimer’s disease (AD) [15, 16], and its downstream signaling pathway has been shown to be involved in the inflammatory response process. Activation of mGluR5 significantly inhibits the inflammatory response of microglia to lipopolysaccharide (LPS) [17]. In addition, the anti-inflammatory effects of mGluR5 agonist are abolished in microglial cultures from mGluR5 knockout mice and also blocked by selective mGluR5 antagonists. The above observations strongly support an essential role of mGluR5 and its signaling in protecting neurons from neuroinflammation, which when lost may contribute to the pathology of PD. Remarkably, previous studies investigated the relationship between α-syn and mGluR5. In PD or dementia with Lewy body (DLB) patients, or α-syn transgenic models, the accumulation of α-syn resulting in alterations of mGluR5, as well as interfering with mGluR5 trafficking, showed that mGluR5 plays a crucial role in α-synucleinopathies [18]. Also, the vulnerability of hippocampal neurons to the toxicity of α-syn and Aβ may be mediated via mGluR5 [19]. Together, these results suggest that the regulation of mGluR5 might play an essential role in the α-syn-induced pathogenesis of neuroinflammation and neurotoxicity in PD.

To examine the role and mechanism for mGluR5 on α-syn-induced microglia inflammation, we activated microglia by overexpressing α-syn, and found that activation of mGluR5 partially inhibited α-syn-induced inflammatory response. Furthermore, α-syn facilitated mGluR5 degradation via the lysosomal pathway by binding to it, which also occurred in PD animal models. These novel findings provide insight into an unappreciated mechanism by which mGluR5 controls α-syn-induced microglia inflammation and contributes to PD pathogenesis.

## Methods

### Cell cultures

BV2 murine microglial cells (provided by Professor Xiao Min Wang, Capital Medical University, Beijing, China) were cultured in Dulbecco’s modified Eagle’s medium (DMEM)/F12 (Corning, Manassas, VA, USA) supplemented with 10% fetal bovine serum (FBS, Corning, Manassas, VA, USA) and 1% penicillin/streptomycin (Life Technologies, Carlsbad, CA, USA) at 37 °C and 5% CO_2_.

Human embryonic kidney (HEK) 293T cells were cultured in DMEM (Corning, Manassas, VA, USA) supplemented with 10% FBS and 1% penicillin/streptomycin at 37 °C and 5% CO_2_. Cells were prepared for experiments at early passages (< 5) and 80% confluency.

Rat primary microglia were extracted from the whole brains of 0 or 1-day-old Sprague-Dawley rats (from Beijing WeitongLihua Laboratory Animal Center, SCXK 2016-0006, Beijing, China) as previously described [20]. Briefly, brain tissues were freed from meninges and blood vessels on the surface carefully, and were dissociated by gentle trituration with a pipette; then, the cell suspension was filtered through a 40-μm pore nylon strainer. The isolated cells were transferred to a poly-l-lysine (PLL; Sigma-Aldrich, St. Louis, MO, USA)-coated 75-cm^2^ flask and incubated at 37 °C and 5% CO_2_. After 14 days, primary microglia were harvested by shaking the flasks for 2 h at 180 rpm, 37 °C. The purity of the microglial cells was immunolabeled with an antibody against ionized calcium binding adaptor molecule (Iba)-1 (1:500; Wako Pure Chemical Industries, Osaka, Japan); over 98% of the cells were immunopositive.

Rat primary cortical neurons were cultured as previous described [21]. Briefly, surgeries were performed under sodium pentobarbital anesthesia. Primary cortical neurons were extracted from Sprague-Dawley rats and cultured in PLL-coated 6-well plates (1.0 × 10^6^ cells/well) or 96-well plates (1.0 × 10^4^ cells/well) in neurobasal medium (Gibco, Grand Island, NY, USA) with nerve growth factor (10 ng/mL), l-glutamine (1 mM, Invitrogen, Carlsbad, CA, USA), and B27 supplement (Gibco, Grand Island, NY, USA). The purity of the neuronal cells was immunolabeled with an antibody against neuronal nuclei (NeuN, 1:50; Cell Signaling Technology, Danvers, MA, USA); over 95% of the cells were immunopositive.

### Drug treatments

For the activity of mGluR5 stimulation or blocking, CHPG (150 μM; Tocris Biosciences, Ellisville, MO, USA) or MTEP (100 μM; Tocris Biosciences, Ellisville, MO, USA) were utilized on microglia for the indicated time points after α-syn transfection. Microglial cells were serum starved for another 18 h prior to exposure to CHPG or MTEP. LPS (100 ng/mL; Sigma-Aldrich, St. Louis, MO, USA) was applied to trigger α-syn expression and stimulate microglia for 24 h. To block protein synthesis, BV2 cell cultures were treated with cycloheximide (CHX, 2 μg/mL; Sigma-Aldrich, St. Louis, MO, USA) for the indicated time points. The lysosome activity was blocked by treatment with NH_4_Cl (1, 5, 10 mM; Sigma-Aldrich, St. Louis, MO, USA) for 12 h and the proteasome activity was blocked by treatment with MG132 (1, 5, 10 μM; Sigma-Aldrich, St. Louis, MO, USA) for 12 h. Urate (2, 6, 8-trioxy-purine, 200 μM; Sigma-Aldrich, St. Louis, MO, USA) prepared as a 1000× stock solution by dissolving in 1 M NaOH was used for microglia after lentivirus vector (LV)-α-syn infection for 24 h. Recombinant human α-synuclein protein (1 μM; Abcam, Cambridge, MA, USA) was performed in primary microglia.

### Plasmids construction and transfection

The cDNAs of rat FLAG-mGluR5 and FLAG-GFP-mGluR3 were kind gifts from Prof. Jun Qi He (Capital Medical University, Beijing, China). The cDNAs of human pCMV-myc-α-syn (myc-α-syn) and myc-α-syn N terminus (amino acids 1–65, abbreviated to myc-α-syn N) were kindly provided by Prof. Hui Yang (Capital Medical University, Beijing, China). The cDNAs of myc-α-syn N terminus deletion (amino acids 61–140, abbreviated to myc-α-syn delN) were produced by Genechem (Shanghai, China). At approximately 80% confluence, BV2 microglial cells or HEK293T cells were transfected with plasmids using Lipofectamine 3000 reagent (Invitrogen, Carlsbad, CA, USA) followed by further analysis.

### Lentivirus generation and recombinant adeno-associated virus (AAV) construction

Lentivirus generation was utilized for primary microglia. For overexpression of α-syn, the sequence of human α-syn cDNA was cloned into the pLVX-Ubi-EGFP lentiviral vector with BamHI and AgeI restriction sites (Shanghai Genechem, Co., Ltd., China), abbreviated to LV-α-syn. The pLVX-Ubi-EGFP vector was used as the control (abbreviated to LV-NC). The lentivirus titer units were 1.0 × 10^9^ TU/mL. Cells were infected with multiplicity of infection (MOI) 100 after 5 μg/mL polybrene supplement and then were treated with CHPG or MTEP for further analysis.

Recombinant AAV was used in animals. For overexpression of α-syn, the full length of human α-syn mRNA was cloned into the AAV9-CMV-betaGlobin-MCS-P2A-EGFP-SV40 Poly A viral vector with AsisI and MluI restriction enzymes constructed by Vigene Biosciences (Shandong, China). The resulting packaged virus was verified by quantitative real-time PCR detection and the AAV9-CMV-betaGlobin-hSNCA-P2A-EGFP-SV40 Poly A (abbreviated to AAV-α-syn) genome titer was 8.17 × 10^13^ vg/mL and the titer of the control viral AAV9-CMV-betaGlobin-EGFP-SV40 Poly A (abbreviated to AAV-GFP) was 1.02 × 10^14^ vg/mL.

### Cell viability measurement

Viability of primary neurons was detected with the 3-(4, 5-dimethylthiazol-2-yl)-5-(3-carboxymethoxyphenyl)-2-(4-sulfophenyl)-2H-tetrazolium (MTS, Cell Tilter 96 Aqueous Assay; Promega, Madison, WI, USA) assay or Cell Counting Kit-8 (CCK-8) assay (SUDGEN, China). Primary neurons (1.0 × 10^4^ cells/well) were seeded in PLL-coated 96-well plates and cultured for 24 h. After 24 h of conditional medium application, for MTS assay, MTS solution was mixed to the cells followed by incubation for 1.5 h at 37 °C, and the absorbance was measured at 490 nm on a microplate reader (Elx800; Bio-Tek Instruments, Winooski, VT, USA); for CCK-8 assay, 10 μl of CCK-8 reagent was applied into the medium. After reaction for 2 h at 37 °C, the absorbance was measured at 450 nm on a microplate reader. The cell viability was determined according to the absorbance.

### Co-immunoprecipitation and western blotting

Cells or brain tissues were harvested and lysed in lysis buffer (20 mM Tris-HCl, 200 mM NaCl, 2 mM EDTA, 1% Triton X-100, pH 7.4) with 1× protease inhibitor cocktail (Thermo Fisher Scientific, Rockford, IL, USA). Extracts (1000 μg) were pre-cleared with proteinA/G agarose (40 μl/tube; Sigma-Aldrich), and then incubated with anti-α-syn, -FLAG, or -myc antibody with constant rotation at 4 °C overnight. The precipitant was harvested by centrifugation at 10,000×*g* for 2 min and washed 3 times with lysis buffer to remove nonspecific binding proteins followed by elution, and then beads were removed by centrifugation at 10,000×*g* for 2 min and the supernatants were analyzed by western blotting.

For cells protein extraction, cells were washed twice with ice-cold PBS, and lysed in lysis buffer (1 M Tris-HCl [pH 7.4], 5 M NaCl, 10% NP-40, 10% Na-deoxycholate, 100 mM EDTA). For tissues protein extraction, tissues were lysed with RIPA lysis buffer (Solarbio, Beijing, China). For membranous and cytoplasmic protein extraction, after 3.0 × 10^7^ BV2 cells were transfected with α-syn for 48 h, the cytoplasmic and membrane fractions were isolated and collected using the Membrane and Cytosol Protein Extraction kit (Beyotime Institute of Biotechnology, Shanghai, China) followed by western blotting as previously described [22]. Briefly, samples were separated on SDS polyacrylamide gels, transferred to nitrocellulose membranes, and blocked in 5% nonfat milk in Tris-buffered saline (TBS). Membranes were incubated with the following primary antibodies, including β-tubulin (1:1000), glyceraldehyde 3-phosphate dehydrogenase (GAPDH, 1:1000), β-actin (1:1000), normal mouse IgG (1:1000), cyclooxygenase-2 (COX-2, 1:1000), phosphorylated (p-) c-Jun N terminal kinase (JNK, 1:1000), p-extracellular signal-regulated protein kinase (ERK, 1:1000), p-p38 (1:1000), p-NF-κB p65 (1:1000), p-AKT (1:1000), JNK (1:1000), ERK (1:1000), p38 (1:1000), NF-κB p65 (1:1000), and AKT (1:1000) (all from Cell Signaling Technology, Danvers, MA, USA); mGluR5 (1:1000), inducible nitric oxide synthase (iNOS, 1:1000), and TNF-α (1:1000) (all from Abcam, Cambridge, MA, USA); Na^+^-K^+^ ATPase (1:1000, Santa Cruz Biotechnology, Dallas, TX, USA); α-syn (1:500, BD Biosciences, Franklin Lakes, NJ, USA); tyrosine hydroxylase (TH, 1:5000, Sigma-Aldrich, St. Louis, MO, USA); IL-1β (1:400, R&D Systems, Minneapolis, MN, USA), c-Myc (1:2000, Clontech, Mountain View, CA, USA); and FLAG (1:1000, EMD Millipore, Temecula, CA, USA) at 4 °C overnight, washed with Tris-buffered saline containing 0.1% Tween 20, incubated with secondary antibodies (Cell Signaling Technology, Danvers, MA, USA). Protein signals were visualized using enhanced chemiluminescence (Bio-Rad, Hercules, CA, USA), quantified by Image J software (NIH), and analyzed by GraphPad Prism 5.0 software (GraphPad Inc., La Jolla, CA, USA).

### Nitrite level measurement

BV2 cells (4.0 × 10^5^ cells/well) or rat primary microglia (1.0 × 10^6^ cells/well) were cultured in 6-well plates coated with PLL and then incubated for 24 h with the indicated treatment. NO levels in the culture supernatants were determined using a Griess kit (Promega, Madison, WI, USA) according to the manufacturer’s protocol. The absorbance was measured at 540 nm on a microplate reader.

### Enzyme-linked immunosorbent assay (ELISA) measurement

BV2 cells (4.0 × 10^5^ cells/well), rat primary microglia (1.0 × 10^6^ cells/well), and HEK293T cells (4.0 × 10^5^ cells/well) were seeded in PLL-coated 6-well plates and incubated with indicated treatment. TNF-α and prostaglandin E_2_ (PGE_2_) concentrations in the culture medium were measured with ELISA kits (ExCell Bio, Shanghai, China) based on the manufacturer’s procedure. The absorbance at 450 nm and 420 nm was measured for TNF-α and PGE_2_ on a microplate reader, respectively. α-syn level in the culture medium was tested with Human Alpha-synuclein ELISA Kit (Abcam, Cambridge, MA, USA) based on the manufacturer’s procedure. The absorbance at 450 nm was measured on a microplate reader.

### Real-time PCR analysis

Rat brain substantia nigra (SN) section samples were processed for total RNA extraction using Trizol reagent (Invitrogen, Carlsbad, CA, USA). RNA concentration was detected by NanoDrop 2000 (Thermo Fisher Scientific, Rockford, IL, USA), and the quality was checked on agarose gels. One microgram total RNA was used for reverse transcription with ImProm-II Reverse Transcription System (Promega, Madison, WI, USA) in a total volume of 20 μl according to the manufacturer’s procedure. Quantitative SYBR Green PCR measurements for α-syn gene expression were performed by the SYBR FAST qPCR Kit Master Mix (2×) (Kapa Biosystems, Wilmington, MA, USA) with prevalidated primers. For α-syn gene amplification, the forward and reverse primers were 5′-AAGGGTACCCACAAGAGGGA-3′ and 5′-AACTGAGCACTTGTACGCCA-3′, respectively. For housekeeping gene GAPDH, forward and reverse primers were 5′-TGACATCAAGAAGGTGGTGAAGC-3′ and 5′-GGAAGAATGGGAGTTGCTGTTG-3′, respectively. The amplification was performed with 40 cycles of 95 °C for 3 s and 60 °C for 30 s on a CFX96TM real-time PCR detection system (Bio-Rad, Hercules, CA, USA). The fold change of α-syn gene expression was normalized to that of GAPDH and was determined by the 2^−ΔΔCt^ method.

### Animals and treatment

Male Sprague-Dawley rats (Beijing WeitongLihua Laboratory Animal Center, SCXK 2016-0006, Beijing, China) weighing 200–250 g were housed under a 12-h light/dark cycle at 22 ± 2 °C with access to food and water ad libitum, and acclimated for 7 days before experiments. All procedures were performed on the basis of the National Institutes of Health Guide for the Care and Use of Laboratory Animals and were approved by the Committee on Animal Care and Usage (Capital Medical University). The sample sizes used in this study were based on estimations from a power analysis.

For the LPS-induced model, the rats (*n* = 45) were randomly divided into 3 groups: sham group (PBS + intraperitoneal (i.p.) injection of vehicle, *n* = 14), LPS group (LPS + i.p. injection of vehicle, *n* = 15), and LPS + urate group (LPS + i.p. injection of urate, *n* = 16). Rats received stereotaxic injection of LPS (Sigma-Aldrich, 5 μg/μl, dissolved in PBS, for a total dose of 10 μg /300 g) and i.p. injection with 200 mg/kg urate (40 mg/mL, dissolved in 0.9% NaCl solution) or its vehicle twice per day, with 1 h between two injections [23]. Rats were anesthetized by pentobarbital sodium i.p. injection and positioned in a stereotaxic apparatus. LPS was injected into the right SN (AP = − 5.5; ML = − 1.5; DV = − 8.3) at a rate of 0.5 μl/min. The needle was remained in place for over 5 min before slow retraction to prevent reflux along the injection tract. The mortality rate of rats in the LPS injection group was < 10%. Rats were killed by anesthetization with pentobarbital sodium followed by decapitation 4 weeks after LPS administration for further analysis.

For the AAV-α-syn-induced model, to observe the progression of PD degeneration with α-syn overexpression, the animals (*n* = 100) were divided into 2 groups: AAV-GFP (AAV-GFP virus vectors + i.p. injection of vehicle, *n* = 50) and AAV-α-syn (AAV-α-syn virus vectors + i.p. injection of vehicle, *n* = 50). To further investigate the mGluR5 mediation on the α-syn-induced inflammatory effect, the animals (*n* = 60) were divided into 5 groups: AAV-GFP (*n* = 10), AAV-α-syn (*n* = 10), AAV-α-syn + MTEP (AAV-α-syn virus vectors + i.p. injection of MTEP, *n* = 15), AAV-α-syn + urate (AAV-α-syn virus vectors + i.p. injection of urate, *n* = 15), and AAV-GFP + urate (AAV-GFP virus vectors + i.p. injection of urate, *n* = 10). The rats were deeply anesthetized by intraperitoneal injection of pentobarbital sodium and fixed in a stereotaxic apparatus. Animals were injected with 3 μl of viral solution (AAV-GFP or AAV-α-syn) into the right SN pars compacta (AP = − 5.5; ML = − 1.5; DV = − 8.3) at a flow rate of 0.2 μl/min through the hole in the skull. The needle was kept for an additional 5 min before withdrawal. In AAV-α-syn + MTEP group, animals received MTEP (1.5 mg/kg/day) i.p. injection at 1 week before virus injection for continuous 3 weeks. In AAV-α-syn + urate group or AAV-GFP + urate group, rats received i.p. injection with 200 mg/kg urate (40 mg/mL, dissolved in 0.9% NaCl solution) or vehicle twice per day, with 1 h between two injections. The death rate after surgery was < 10%. After the indicated time of virus delivery, the animals were sacrificed and brain tissues were harvested for further analysis.

### Behavioral tests

Rats were tested at indicated periods post-surgery with the rotation test, rota-rod behavioral test, and open field test. In apomorphine (Sigma-Aldrich)-induced rotation behavior test, the rotational rate (number of rotations during the 30-min testing period divided by 30) was calculated. Motor function was determined by a rota-rod treadmill for rats under an accelerating rotor mode. Animals training consisted of four trials daily for 3 days (with 15 min rests between trials). Rats were placed on the rotating rod at 5 rpm and accelerating gradually to 30 rpm for 5 min, and the interval times from the beginning until rats fell off was recorded as performance time. The performance on the rota-rod test was measured 5 times per rat. Open field test was also used to judge locomotor activity as previously described [24, 25]. Briefly, the equipment consisted of a 100 cm × 100 cm × 100 cm square arena that was divided into 9 equal squares. A single rat was placed in the center of the square, and the distance of traveling in the area of the apparatus was counted for 30 min.

### Immunohistochemistry and immunofluorescence

Rats were deeply anesthetized with pentobarbital sodium and transcardially perfused with saline followed by 4% paraformaldehyde (PFA, Sigma-Aldrich, St. Louis, MO, USA) dissolving in 0.1 M PBS (pH 7.4). The brains were dehydrated in 20% and 30% sucrose solutions and then coronally sectioned at 40-μm thickness on a freezing microtome (Leica, Solms, Germany). For the immunohistochemistry, sections in SN and striatum (STR) regions were incubated with 3% hydrogen peroxide for 10 min to block endogenous peroxidase activity. After washing with PBS, sections were permeabilized with 0.3% Triton X-100 in PBS for 30 min followed by incubation with 10% normal horse serum for 1 h at room temperature and then incubated in the primary antibody anti-TH (1:2000, Sigma-Aldrich) overnight at 4 °C. After 3 washes with PBS, biotinylated anti-mouse secondary antibody (Vector Stain ABC kit, Burlingame, CA, USA) and diaminobenzidine (DAB, Zhongshan Golden bridge Biotechnology, Beijing, China) were utilized to visualize immunoreactivity. Consecutive sections for 20 slices were selected from each brain for detection. Unbiased stereology was applied to count the number of TH+ neurons in SN region on a DM5000B microscope (Leica Microsystems, Bannockburn, IL, USA) with Stereo Investigator software (MBF Bioscience, Williston, VT, USA). The intensity of TH+ fibers in the STR was quantified using Image Pro Plus v5.0 image analysis software (Datacell, London, UK).

For immunofluorescence, cultured cells or tissues from SN regions were fixed with 4% PFA for 15 min followed by permeabilization with 0.3% Triton X-100 in PBS for 30 min, and then incubation with 10% normal horse serum for 1 h at room temperature. Primary antibodies were added at 4 °C overnight. After washing in PBS, the sections were incubated with Alexa Fluor 594 donkey anti-rabbit (1:200, Invitrogen), Alexa Fluor 647 donkey anti-goat (1:200, Invitrogen), Alexa Fluor 488 donkey anti-mouse (1:200, Invitrogen), Alexa Fluor 405 donkey anti-rat (1:100, Abcam), Alexa Fluor 488 donkey anti-rabbit (1:200, Invitrogen), and Alexa Fluor 594 donkey anti-mouse (1:200, Invitrogen) for 1 h in the dark. The nuclei were counterstained with 4′,6-diamidino-2-phenylindole (DAPI, Cell Signaling Technology) for 5 min. Primary antibodies included rabbit polyclonal anti-mGluR5 (1:200, EMD Millipore), goat monoclonal anti-Iba-1 (1:200; Abcam), mouse monoclonal anti-α-syn (1:100, BD Biosciences), rat monoclonal anti-lysosome-associated membrane protein (LAMP)-1 (1:50, Santa Cruz Biotechnology), rabbit polyclonal anti-NeuN (1:50; Cell Signaling Technology). and mouse monoclonal anti-TH (1:2000, Sigma-Aldrich). Images were captured using a confocal microscope (TCS SP8; Leica, Solms, Germany).

### Protein-protein modeling simulation

The crystal structures of the protein α-syn for docking process were obtained from the Protein Data Bank database (PDB ID: 2NOA); the 3D (three-dimensional) structures of protein mGluR5 were constructed from ab initio modeling utilizing the software I-TASSER. Protein-protein docking simulations for mGluR5-α-syn complex were based on professional software Rosetta (http://robetta.bakerlab.org/). In docking processes, the protein α-syn and mGluR5 were assigned as “ligand” and “receptor”, respectively, and global scanning of the rotational and translational space were performed. Molecular docking results were scored in Rosetta with the default values of 1-Å grid step and 4-Å surface-layers used for clustering. Finally, a maximum number of 50 conformers were considered, and the conformation with the lowest binding energy was selected for final molecular dynamics simulations.

Molecular dynamics simulations were performed by using the AMBER16 package (http://ambermd.org/) with default scoring parameters utilizing the GAFF and AMBERff 14SB force field. All the simulations were carried out at constant temperature (310 K) and pressure (1 bar), and simulations modeling results were recorded at each 2 fs time-scale step. Van der Waals energy and short range electrostatic energy calculations radius cutoff were set as 10 Å, and the long-range electrostatic energy was calculated with default configurations by the PME method.

### Statistical analysis

Data are expressed as the mean ± SD or mean ± SEM and analyzed using Prism 5.0 software (GraphPad Inc., La Jolla, CA, USA). Where parametric tests were used, we checked normal distribution and difference in variance by the Shapiro-Wilk test and an *F* test, respectively. A two sample unpaired Student’s *t* test was used for two-group comparisons. One-way ANOVA followed by Dunnett’s test was used for multiple-group comparisons. Two-way repeated ANOVA was used to determine the statistical changes in each measure. At least three independent experiments were performed for each assay. *p* < 0.05 were considered significant throughout the study.

## Results

### mGluR5 interacted with α-syn in microglia

**Fig. 1:**
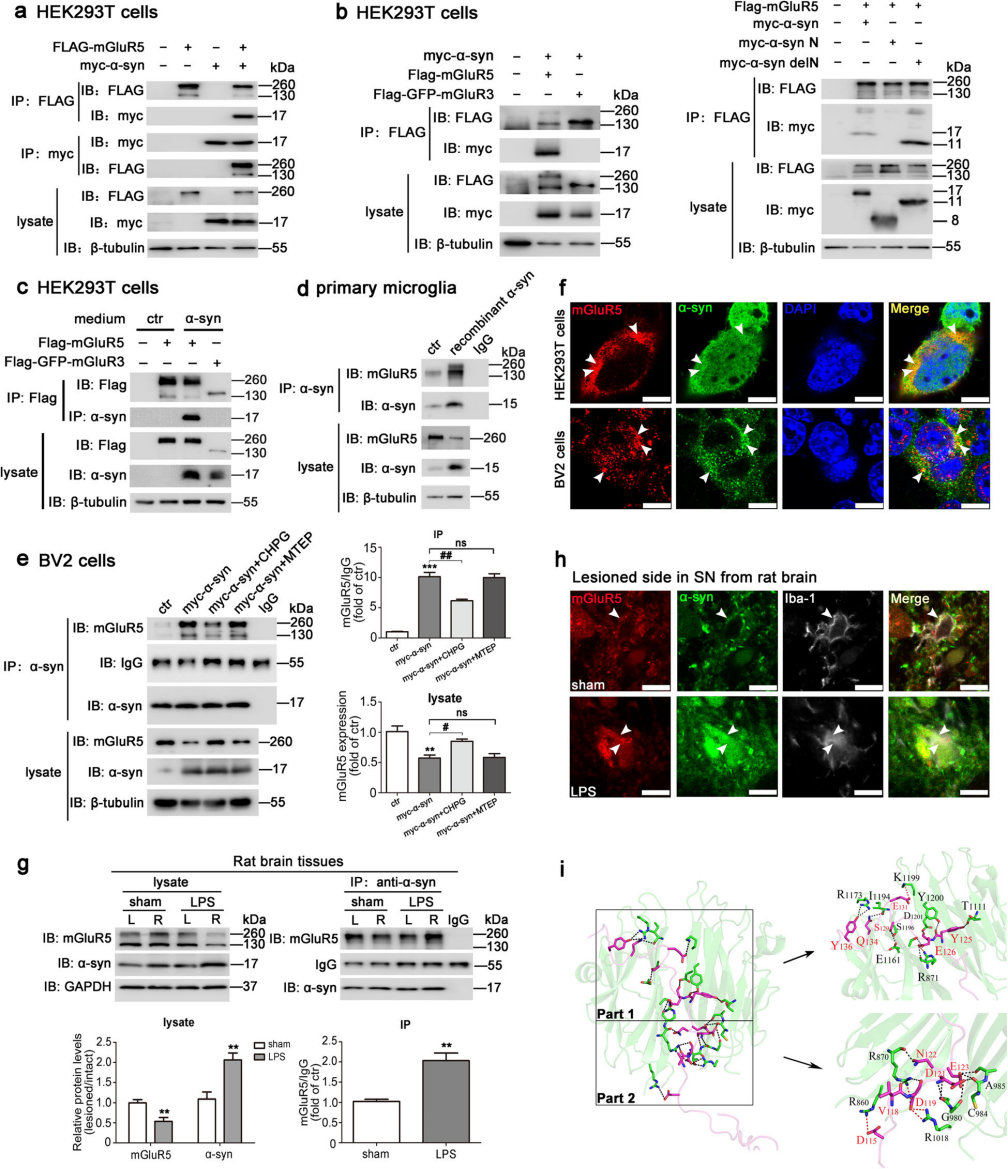
mGluR5 interacted with α-syn in microglia. **a** Interaction of mGluR5 and α-syn detected by immunoprecipitation with antibodies against myc and FLAG in co-transfected HEK293T cells. **b** HEK293T cells were co-transfected with myc-α-syn, and FLAG-mGluR5 or FLAG-GFP-mGluR3 plasmids; the specificity of mGluR5 interaction with α-syn was detected by co-immunoprecipitation (left). HEK293T cells were co-transfected with FLAG-mGluR5, and full-length myc-α-syn, myc-α-syn N, or myc-α-syn delN plasmids; mGluR5 interaction with α-syn truncations was detected by co-immunoprecipitation (right). **c** FLAG-mGluR5 or FLAG-GFP-mGluR3 overexpressed HEK293T cells were cultured with the collected medium from myc-α-syn-overexpressed HEK293T cells for 12 h, followed by co-immunoprecipitation. **d** Primary microglia were treated with 1 μM recombinant α-syn protein for 24 h, followed by co-immunoprecipitation. **e** BV2 cells were transfected with myc-α-syn for 24 h followed by CHPG (150 μM) or MTEP (100 μM) treatment for 24 h. Co-immunoprecipitation was performed to detect the interaction between mGluR5 and α-syn (left). Quantification of immunoprecipited mGluR5 level was normalized to IgG, and protein levels of mGluR5 and α-syn in cell lysates were normalized to β-tubulin (right). Cells transfected with vector without CHPG treatment served as control. **f** Immunofluorescence analysis was conducted from HEK293T cells co-transfected with myc-α-syn and FLAG-mGluR5 for 48 h (top), or BV2 cells transfected with myc-α-syn for 48 h (bottom). Cells were fixed and stained with anti-mGluR5 (red) and anti-α-syn (green), and nuclei were stained with DAPI. Arrowheads indicate examples of co-localization. Scale bar, 10 μm. **g** Brain lysates from LPS-induced rat PD model were immunoprecipitated with control mouse IgG or anti-α-syn, and the coprecipitated proteins were analyzed by immunoblotting with anti-mGluR5 (top). Quantification of protein levels of mGluR5 and α-syn in lysates were normalized to GAPDH and represented as the fold different ratio of the sham group. Immunoprecipited mGluR5 level was normalized to IgG and represented as the fold different ratio (lesioned/intact) of the sham group (bottom). **h** Immunofluorescent co-stainings with anti-mGluR5 (red) and anti-α-syn (green) were conducted with brain sections of SN in sham group (top) and LPS group (bottom). Microglia were stained with anti-Iba-1 (gray). Arrowheads indicate examples of co-localization. Scale bar, 10 μm. **i** Structural view of the representative mGluR5-α-syn complex predicted by protein-protein docking. The left showed the complex structure, and mGluR5 was colored in green and α-syn was colored in red. The right showed the predicted key point residues from the region of mGluR5 and the α-syn in each part. The residues in mGluR5 were colored in black, and the residues in α-syn were colored in red. Hydrogen bonds were shown as black dashed lines, and salt bridges were shown as red dashed lines, also represented the stronger interaction between five key point residues on intracellular C-terminal domain of mGluR5 and four key point residues from C-terminal region of α-syn indicated above. Carbon atoms, oxygen atoms, and nitrogen atoms for both mGluR5 and α-syn were colored in green, red, and blue, respectively. Ribbon were colored green for mGluR5 and colored red for α-syn. Data shown in this figure represent mean ± SEM (**e**, *n* ≥ 3) and mean ± SD (**g**, *n* ≥ 6). The statistical significance was determined by one-way ANOVA followed by Dunnett’s test (**e**). A comparison of mGluR5 expression in co-immunoprecipitation studies between sham group and LPS group using Student’s *t* test (**g**). ^**^p < 0.01 and ^***^p < 0.001 versus control (ctr) or sham group; ^#^*p* < 0.05 and ^##^*p* < 0.01 versus α-synuclein (α-syn) transfection; ns, not significant

The alterations in the levels of mGluR5 in selected brain regions display increased α-syn accumulation in patients with DLB or PD, as well as in α-syn transgenic mice; thus, the vulnerability of hippocampal neurons to α-syn and Aβ might be mediated by mGluR5 [18, 19]. It has been also reported that both α-syn and mGluR5 play roles in microglia activation [9, 17]. We therefore hypothesized that there might be a crosslink between mGluR5 and α-syn in microglia. We first co-transfected FLAG-tagged mGluR5 and myc-tagged α-syn using HEK293T cells as an experimental tool to directly examine the interaction between mGluR5 and α-syn. mGluR5 immunoprecipitation with an anti-FLAG antibody was followed by western blotting analysis of α-syn. It showed the robust co-immunoprecipitation of α-syn with mGluR5. FLAG-tagged mGluR5 was also observed in the immunoprecipitated complex with an anti-myc antibody (Fig. 1a). To further characterize the association between α-syn and mGluR5, one of the metabotropic glutamate receptors (mGluR3) was tested for binding detection. The results showed that α-syn selectively interacted with mGluR5 but not with mGluR3 in co-transfected HEK293T cells (Fig. 1b, left). The α-syn N-terminus, which adopts an α-helical conformation upon lipid binding, is essential for membrane interaction [26], and mGluR5, as a G protein coupled receptor, can be trafficked to the plasma membrane [27]. To test whether the N-terminus of α-syn was essential for the interaction between these two proteins, myc-α-syn N or myc-α-syn delN constructs were overexpressed with mGluR5 in HEK293T cells, followed by a co-immunoprecipitation assay. The truncation assay showed that α-syn delN, but not the N-terminus of α-syn, was associated with mGluR5 (Fig. 1b, right). To further test whether extracellular α-syn taken up by cells could interact with mGluR5 intracellularly, we treated FLAG-mGluR5 or FLAG-GFP-mGluR3 overexpressed HEK293T cells with the collected medium from myc-α-syn-overexpressed HEK293T cells, followed by a co-immunoprecipitation assay. As expected, α-syn overexpression in HEK293T cells caused a significant increased level of α-syn in the culture medium (Additional file 1: Figure S1), and then the released α-syn could be engulfed by mGluR5 or mGluR3-overexpressed HEK293T cells, leading to the α-syn complex formed in mix-cultured cell system with mGluR5 but not with mGluR3 (Fig. 1c). To further confirm that mGluR5 interacted with α-syn in microglia, primary microglia were treated with 1 μM recombinant human α-syn protein, and co-immunoprecipitation revealed that the engulfed exogenous α-syn interacted with the endogenous mGluR5 in microglia (Fig. 1d). Moreover, we performed an α-syn immunoprecipitation assay followed by western blotting analysis for mGluR5 in BV2 microglial cells overexpressing α-syn, showed that mGluR5, stimulated by the receptor agonist CHPG, and weakened the interaction with α-syn. However, MTEP, a potent and selective mGluR5 antagonist, had little effect on the binding of these two proteins, while CHPG treatment increased the protein levels of mGluR5 in cell lysates, suggesting that activation of mGluR5 reduces the binding to α-syn, to affect the protein levels of mGluR5 receptors (Fig. 1e). Further evidence for a cellular mGluR5-α-syn complex was obtained from double immunofluorescence studies that showed the co-localization of mGluR5 with α-syn in the cytoplasm of HEK293T cells or BV2 microglial cells (Fig. 1f). Importantly, as an LPS-induced PD model is considered to mainly cause microglia inflammation [28], we performed a co-immunoprecipitation assay to show that the binding between mGluR5 and α-syn was enhanced in rat SN sections where LPS was injected, compared with the sham group. It suggests that the interaction between these two proteins in microglia exerted a stronger function pathologically, which also seems likely to be physiologically relevant. In addition, we found that the expression level of mGluR5 was decreased by 0.46-fold (*p* = 0.0032) and the expression level of α-syn was increased by 0.89-fold (*p* = 0.0023) in LPS-treated lysates (Fig. 1g). The immunofluorescent staining further showed the co-localization of these two proteins in microglia labeled with an anti-Iba-1 antibody (Fig. 1h). Based on these results, a three-dimensional model of the mGluR5-α-syn complex predicted by protein-protein docking studies was conducted, which showed the amino acid residues were involved in the interaction interface between mGluR5 and α-syn. According to the number of hydrogen bonds and salt bridges (Additional file 2: Table S) and the complementarity of hydrophobic/hydrophilic properties on the mGluR5 and α-syn molecular surfaces (Fig. 1i), five key point residues on intracellular C-terminal domain of mGluR5 (K1199, R871, R860, R870, and R1018) and four key point residues on the C-terminal region of α-syn (E131, E126, D115, and D119) were identified by a computational modeling method that characterized the interaction between mGluR5 and α-syn. The predicted mGluR5-α-syn binding pattern together with the experimental data indicated that the C-terminal of α-syn might be the key domain that combined with mGluR5 on its C-terminal domain. Together, our results showed that α-syn interacted with mGluR5, which was strengthened by LPS stimulation, suggesting that mGluR5 could regulate α-syn-induced inflammation via the interaction with α-syn.

### Activation of mGluR5 inhibited α-syn-induced inflammatory signaling

**Fig. 2:**
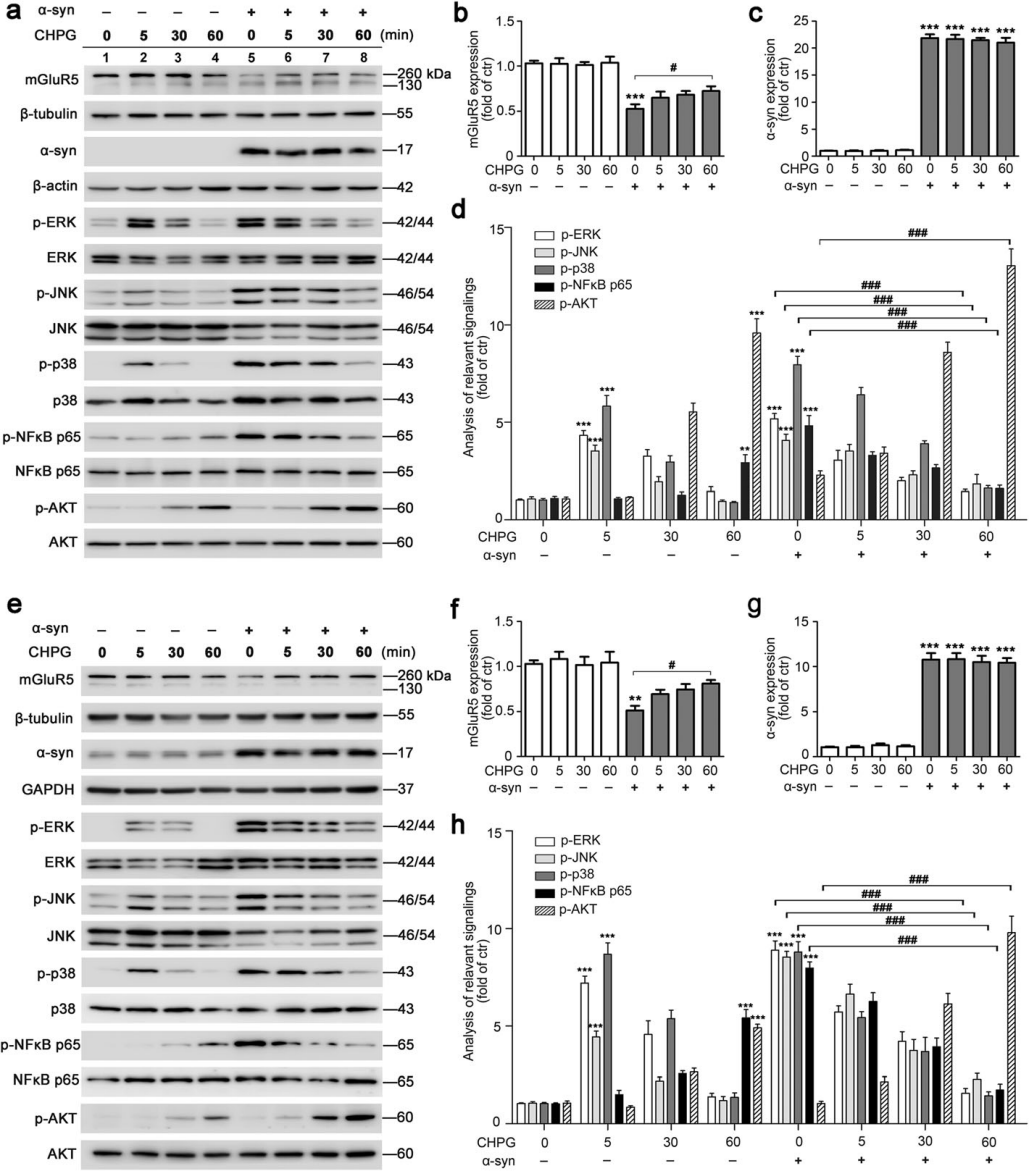
Activation of mGluR5 inhibited α-syn-induced inflammatory signaling. **a** BV2 cells were transfected with myc-α-syn or vector for 36 h, followed by treatment with CHPG (150 μM) for the indicated time points. The expression of mGluR5, α-syn, and the activities of ERK, JNK, p38, NF-κB p65, and AKT were detected by western blotting. **b**–**d** Protein levels of mGluR5 and α-syn were normalized to β-tubulin and β-actin, respectively (**b**, **c**). The p-ERK, p-JNK, p-p38, p-NF-κB p65, and p-AKT levels were normalized to the total of ERK, JNK, p38, NF-κB p65, and AKT, respectively (**d**). Cells transfected with vector without CHPG treatment served as control (**a**–**d**). **e** Primary microglia were infected with LV-NC or LV-α-syn followed by CHPG (150 μM) for different time periods. The expression of mGluR5, α-syn, and the activities of ERK, JNK, p38, NF-κB p65, and AKT were detected by western blotting. **f**–**h** Protein levels of mGluR5 and α-syn were normalized to β-tubulin and GAPDH, respectively (**f**, **g**). The p-ERK, p-JNK, p-p38, p-NF-κB p65, and p-AKT levels were normalized to the total of ERK, JNK, p38, NF-κB p65, and AKT, respectively (**h**). Cells infected with LV-NC without CHPG treatment served as control (**e**–**h**). Data shown in this figure represent the mean ± SEM (*n* ≥ 3). The statistical significance was determined by one-way ANOVA followed by Dunnett’s test. **p < 0.01 and ***p < 0.001 versus control (ctr); ^#^*p* < 0.05 and ^###^*p* < 0.001 versus the α-synuclein (α-syn) transfection or LV-α-syn infection without CHPG treatment

mGluR5 has been known to modulate MAPKs signaling pathways [29]. The NF-κB pathway is another downstream signaling pathway of mGluR5, and has also been shown to play a crucial role in α-syn-induced microglial inflammation [30]. Thus, we next examined whether activation of mGluR5 mediated α-syn-induced inflammatory signaling. BV2 cells were transfected with either vector or α-syn for 36 h, and serum starved for another 18 h followed by CHPG stimulation for 0, 5, 30, or 60 min. The phosphorylation of MAPKs, NF-κB, and AKT was assessed by western blotting. The rationale to do so is that long-term starvation decreases the serum-stimulated signaling cascade activity to the lowest basal levels [31], which renders the cells more sensitive to stimulations than the normal condition and thus makes the stimulated effects easier to observe. Figure 2a–d showed that CHPG activated ERK, JNK, and p38, reaching a peak at 5 min, and NF-κB p65 phosphorylation increased in a time-dependent manner and then reached a peak at 60 min, consistent with previous reports [30, 32–34]. In addition, overexpressed α-syn reduced the protein levels of mGluR5 by 0.49-fold (*p* < 0.0001) (Fig. 2a, lane 5 versus lane 1), which was increased by CHPG treatment, consistent with the results shown in Fig. 1e. The inflammatory downstream signaling induced by α-syn administration was blocked in a time-dependent manner when CHPG activated mGluR5; however, the AKT signaling pathway induced by α-syn with CHPG treatment was more potent (Fig. 2a, lanes 6–8 versus 5). One possible explanation for this observation was that the AKT pathway might play a stronger protective role in cell survival in response to α-syn-induced inflammation. The above results were confirmed in primary microglia, in which CHPG prevented the inflammatory signaling pathway stimulated by LV-α-syn infection (Fig. 2e–h). Notably, mGluR5 activation by CHPG treatment increased phosphorylation of NF-κB p65, and MAPK signaling pathway proteins; however, α-syn overexpression also induced these signaling pathway proteins, which was downregulated by mGluR5 activation. These results implied a crosslink of signaling pathways between mGluR5 and the inflammation in microglia, showing different regulations of mGluR5 with or without α-syn-induced inflammation. Taken together, the data indicated that activation of mGluR5 inhibited inflammatory signaling caused by α-syn.

### Activation of mGluR5 partially inhibited microglia activation induced by α-syn

**Fig. 3:**
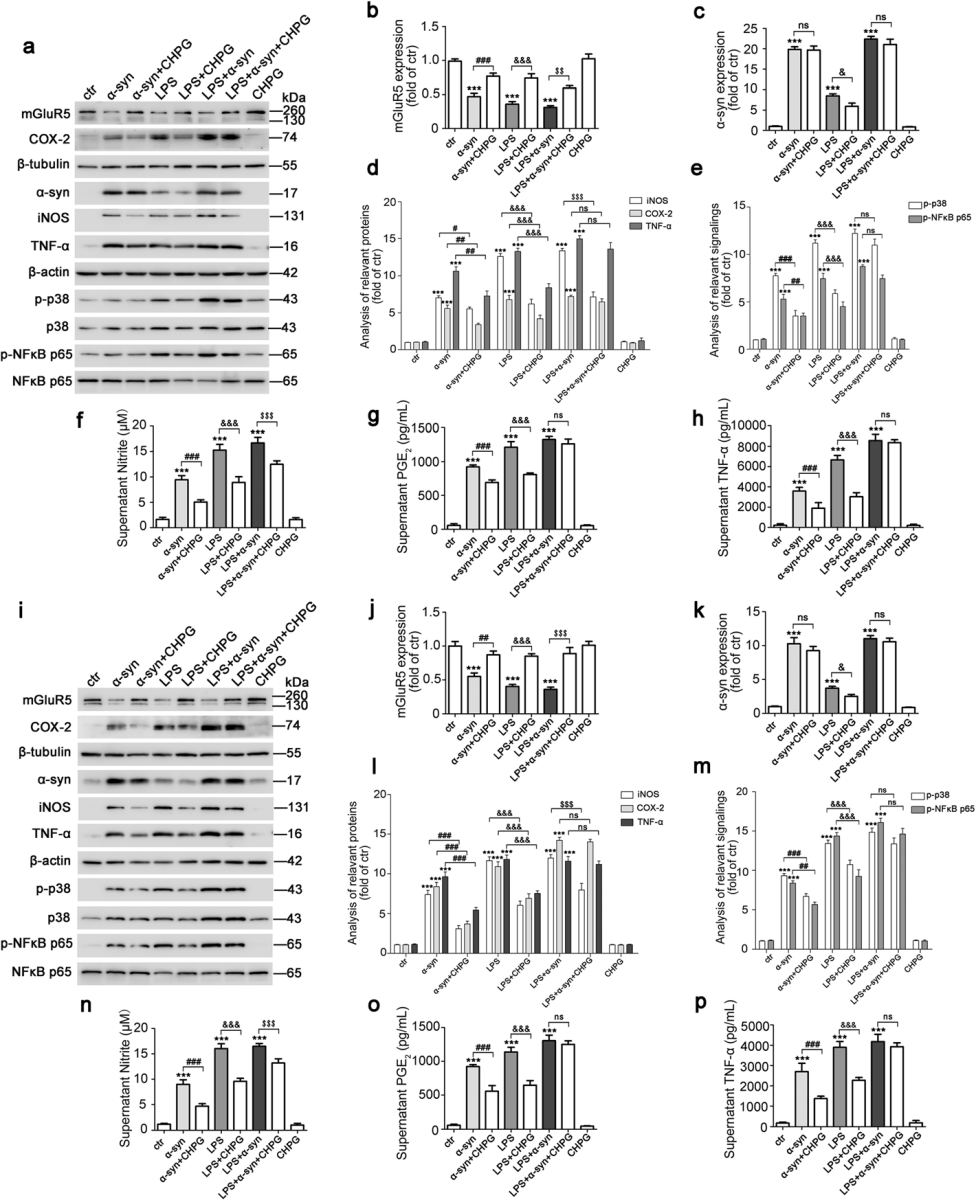
Activation of mGluR5 partially inhibited microglia activation induced by α-syn. **a** BV2 cells were transfected with myc-α-syn or vector for 24 h followed by CHPG (150 μM) administration for 24 h, followed by pre-treatment with CHPG for 1 h before LPS (100 ng/mL) treatment for 24 h, or followed by combination with these two applications. Immunoblotting analysis of cell lysates was detected by various antibodies. **b**–**e** Quantification of protein bands was normalized to β-tubulin (mGluR5, COX-2) and β-actin (α-syn, iNOS, TNF-α) (**b**–**d**). The p-p38 and p-NF-κB p65 levels were normalized to total p38 and NF-κB p65, respectively (**e**). **f**–**h** Culture supernatants from BV2 cells were assayed for NO (**f**), PGE_2_ (**g**), and TNF-α (**h**) release by ELISA. Cells transfected with vector without drugs administration served as control (**a**–**h**). **i** Primary microglia were infected with LV-NC or LV-α-syn followed by CHPG (150 μM) administration for 24 h, followed by pre-treated with CHPG for 1 h before LPS (10 ng/mL) treatment for 24 h, or followed by combination with these two treatments. Cell lysates were probed by using indicated specific antibodies. **j**–**m** Quantification of protein bands was normalized to β-tubulin (mGluR5, COX-2) and β-actin (α-syn, iNOS, TNF-α) (**j**–**l**). The p-p38 and p-NF-κB p65 levels were normalized to total p38 and NF-κB p65, respectively (**m**). **n**–**p** Culture supernatants from primary microglia were assessed for NO (**n**), PGE_2_ (**o**), and TNF-α (**p**) release by ELISA. Cells infected with LV-NC without drugs treatment served as control (**i**–**p**). Data shown in this figure represent mean ± SEM (*n* ≥ 3). The statistical significance was determined by one-way ANOVA followed by Dunnett’s test. ***p < 0.001 versus control (ctr); ^#^*p* < 0.05, ^##^*p* < 0.01, and ^###^*p* < 0.001 versus α-synuclein (α-syn) transfection or the LV-α-syn infection. ^&^*p* < 0.05 and ^&&&^*p* < 0.001 versus LPS. ^$$^*p* < 0.01 and ^$$$^*p* < 0.001 versus the LPS accompanied by α-syn. ns, not significant

LPS induces microglia activation and increases pro-inflammatory cytokine production in vitro [35–37]. In addition, α-syn protein and mRNA levels in the hippocampus are higher in the LPS intraperitoneal injection group of mice compared with the sham group [38]; we consistently found that brain lysates in the SN with LPS injection had increased α-syn protein level compared with the sham group (Fig. 1g). Based on this effect, we then examined whether activation of mGluR5 mediated the biological effect of α-syn on inflammation. BV2 cells were transfected with α-syn or empty vector for 24 h, followed by CHPG treatment and/or LPS treatment for 24 h. This particular paradigm was applied in order to allow exposure to treatments for a reasonable time to induce and accumulate effects. The results showed that the level of mGluR5 expression was decreased when α-syn was transfected or/and cells were stimulated by LPS, while CHPG treatment effectively reversed this process (Fig. 3a, b); correspondingly, LPS treatment increased the level of α-syn expression by approximately 7.6-fold (*p* < 0.0001) (Fig. 3a, c). Microglia activation increases the expression level of chemokines and released classical neurotransmitters such as pro-inflammatory cytokines, TNF-α, and NO, which is produced by iNOS [39], and PGE_2_, which is biosynthesized by the rate-limiting enzyme, COX-2. Our results also consistently showed that α-syn overexpression increased the protein levels of TNF-α, iNOS, and COX-2, and this inflammatory response was stronger during LPS treatment together with overexpressed α-syn. As expected, activation of mGluR5 by treatment with CHPG partially suppressed α-syn-induced inflammation. Notably, the inflammatory response to activation of mGluR5 was not significantly affected, but iNOS expression showed a 0.47-fold decrease (*p* < 0.0001) by CHPG administration when cells were exposed to LPS treatment accompanied by α-syn transfection, suggesting that mechanisms other than mGluR5 activation may have been involved in the synergistic effect of LPS and α-syn (Fig. 3a, d). Correspondingly, the released levels of pro-inflammatory factors such as NO, PGE_2_, or TNF-α were significantly upregulated by α-syn overexpression and/or LPS treatment, whereas CHPG partially inhibited these pro-inflammatory factors, and the synergistic effect of LPS and α-syn on mGluR5 was not significantly affected by CHPG except NO production (Fig. 3f–h). To confirm these results, we also tested classical inflammation signaling involving the phosphorylation of p38 and NF-κB p65. In agreement with the results shown in Fig. 2, CHPG inhibited these processes when either α-syn was overexpressed or LPS was used for treatment (Fig. 3a, e). In addition, the anti-inflammatory effect of mGluR5 regulation on α-syn was confirmed in primary microglia (Fig. 3i–p). Taken together, these results indicated that activation of mGluR5-mediated signaling partially inhibited microglia activation induced by α-syn.

### Overexpression of α-syn decreased mGluR5 expression via lysosomal degradation

**Fig. 4:**
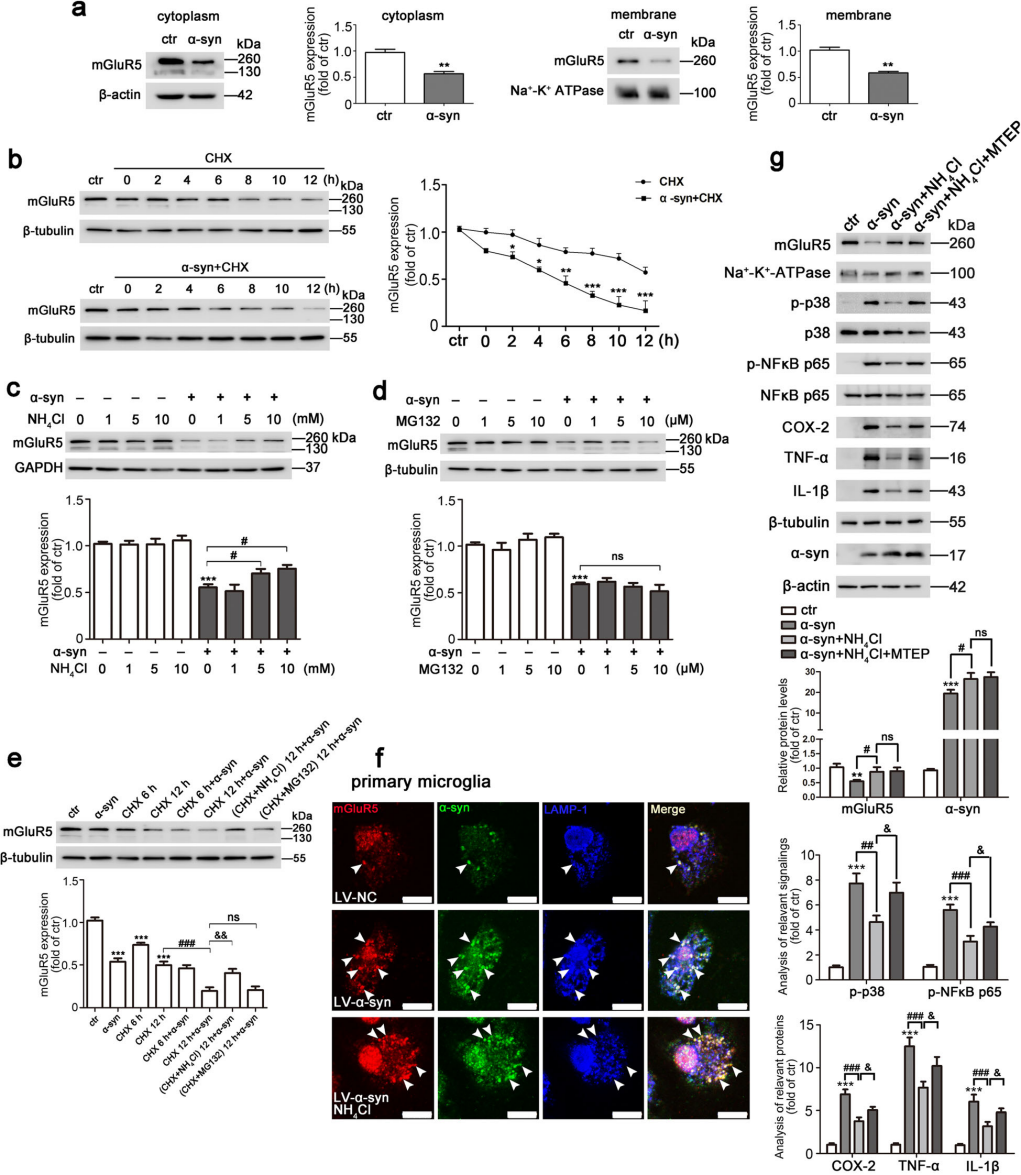
α-syn regulated mGluR5 degradation by lysosomes. **a** BV2 cells were transfected with myc-α-syn or vector for 48 h. mGluR5 expression in the cytoplasmic fraction was evaluated by western blotting, and quantification of mGluR5 level was normalized to β-actin (left). mGluR5 expression in the membrane fraction was evaluated by western blotting, and quantification of mGluR5 level was normalized to Na^+^-K^+^-ATPase (right). Cells transfected with vector served as control. **p < 0.01 versus control. **b** Western blotting analysis of mGluR5 levels under CHX (2 μg/mL) exposure for 0–12 h for the absence or presence of α-syn in BV2 cells. Quantification of mGluR5 levels was normalized to β-tubulin. Cells transfected with vector without CHX treatment served as control. *p < 0.05, **p < 0.01, and ***p < 0.001 versus CHX group. **c**, **d** BV2 cells were transfected with myc-α-syn or vector for 24 h followed by treatment with NH_4_Cl (1, 5, 10 mM) (**c**) or MG132 (1, 5, 10 μM) (**d**) for 12 h, and mGluR5 expression was detected by western blotting. Quantification of mGluR5 levels was normalized to GAPDH (**c**) and β-tubulin (**d**). Cells transfected with vector without drugs administration served as control. ***p < 0.001 versus control; ^#^*p* < 0.05 versus α-syn transfection. **e** BV2 cells were transfected with myc-α-syn or vector for 24 h followed by treatment with NH_4_Cl (10 mM) or MG132 (10 μM), accompanied by CHX (2 μg/mL) treatment for 6 h or 12 h, and mGluR5 expression was detected by western blotting (top). Quantification of mGluR5 level was normalized to β-tubulin (bottom). Cells transfected with vector without drugs treatment served as control. ***p < 0.001 versus control, ^###^*p* < 0.001 versus CHX treatment for 12 h, ^&&^*p* < 0.01 versus α-syn transfection with CHX treatment for 12 h. **f** Immunofluorescent co-stainings with anti-mGluR5 (red), anti-α-syn (green), and anti-LAMP-1 (blue) were conducted in primary microglia infected with LV-NC, LV-α-syn, or LV-α-syn with NH_4_Cl treatment. Arrowheads indicate examples of co-localization. Scale bar, 10 μm. **g** BV2 cells were transfected with myc-α-syn or vector for 24 h followed by treatment with NH_4_Cl (10 mM) or NH_4_Cl combined with MTEP (100 μM) for 12 h. Immunoblotting analysis of membrane fraction with an anti-mGluR5 antibody was conducted, and cell lysates with various antibodies were also detected (top). Quantification of protein bands was normalized to Na^+^-K^+^-ATPase (mGluR5), β-tubulin (COX-2, TNF-α, IL-1β), and β-actin (α-syn). The p-p38 and p-NF-κB p65 levels were normalized to total p38 and NF-κB p65, respectively (bottom). Cells transfected with vector without drugs administration served as control. **p < 0.01, ***p < 0.001 versus control; ^#^*p* < 0.05, ^##^*p* < 0.01, and ^###^*p* < 0.001 versus α-syn transfection; ^&^*p* < 0.05 versus α-syn transfection with MTEP treatment. Ctr, control; α-syn, α-synuclein. Data shown in this figure represent mean ± SEM (*n* ≥ 3). The statistical significance was determined by Student’s *t* test (**a**), two-way ANOVA (**b**), and one-way ANOVA followed by Dunnett’s test (**c**–**e**, **g**). ns, not significant

As discussed above, transfected α-syn in BV2 cells markedly decreased mGluR5 expression levels, which could be reversed by CHPG administration (Figs. 2 and 3). We then investigated how mGluR5 expression was affected by α-syn. To examine whether the change in mGluR5 levels with α-syn overexpression was intracellular or/and on the cell surface, the cytoplasmic and membrane fractions of transfected BV2 cells were extracted for analysis of mGluR5 levels by western blotting. The expression of mGluR5 in the cytoplasm exhibited a 0.42-fold decrease (*p* = 0.0061) by α-syn transfection, and α-syn also significantly reduced mGluR5 levels by 0.43-fold (*p* = 0.0019) on the cell membrane (Fig. 4a), which was similar to the results obtained in whole cells (Fig. 2a). We next analyzed the effect of α-syn on mGluR5 degradation. CHX is a eukaryote protein synthesis inhibitor, which blocks translational elongation by interfering with the translocation step of protein synthesis. Cells were transfected with or without α-syn followed by CHX treatment, and the remaining mGluR5 was detected at different time points. Compared with that in the CHX-treated group, the decreased level of mGluR5 was accelerated by α-syn transfection (Fig. 4b). These results suggested that α-syn promoted the degradation of mGluR5 protein. We further investigated the degradation pathway by which α-syn affected the levels of mGluR5 protein. Figure 4c and d showed that the lysosomal inhibitor, NH_4_Cl, rescued the α-syn-induced degradation of mGluR5 protein by 0.27-fold (*p* = 0.0449) and 0.37-fold (*p* = 0.0106) in a dose-dependent manner of 5 mM and 10 mM, respectively; however, in the presence of the proteasome inhibitor, MG132, the decreased level of mGluR5 by α-syn overexpression had little effect (*p* = 0.3307). Consistent with these results, in the presence of CHX inhibition when cells were transfected with α-syn, NH_4_Cl rescued the remaining mGluR5 degradation induced by α-syn as compared to α-syn accompanied by CHX treatment, but not with MG132 (Fig. 4e). Accordingly, with immunofluorescence staining, the microscopic images revealed that mGluR5 was present more in LAMP-1-positive lysosomes accompanied by co-localizing with α-syn in LV-α-syn-treated primary microglia, as shown that more white dots merged with LAMP-1 staining, but the complex failed to localize in lysosomes with NH_4_Cl administration, as shown that more yellow dots were present when lysosomal function was impaired (Fig. 4f). To further explore the biological effect of mGluR5 when lysosomal function was blocked, we detected the inflammatory response as shown in Fig. 4g. As expected, the level of mGluR5 on the plasma membrane was increased with NH_4_Cl treatment, which reversed the inflammatory signaling such as p-p38 and p-NFκB, causing a significantly decreased inflammatory response. However, MTEP treatment attenuated the anti-inflammatory effects of mGluR5 caused by NH_4_Cl administration. This result indicated that lysosomal impairment reversed the level of mGluR5 on the plasma membrane, and subsequently mGluR5-mediated signaling exerted the anti-inflammatory effects. Notably, lysosomal impairment also led to α-syn accumulation intracellularly, which may cause more severe inflammation. However, we found that the combination of both produced a net anti-inflammatory response with NH_4_Cl treatment, suggesting that mGluR5 is essential for counteracting microglia inflammation induced by α-syn. Together, the results indicated that mGluR5 was trafficked by overexpressed α-syn to the lysosomal compartments for degradation contributing to microglia activation.

### Activation of mGluR5 partially protected neurons from neurotoxicity caused by α-syn-induced microglia activation

**Fig. 5:**
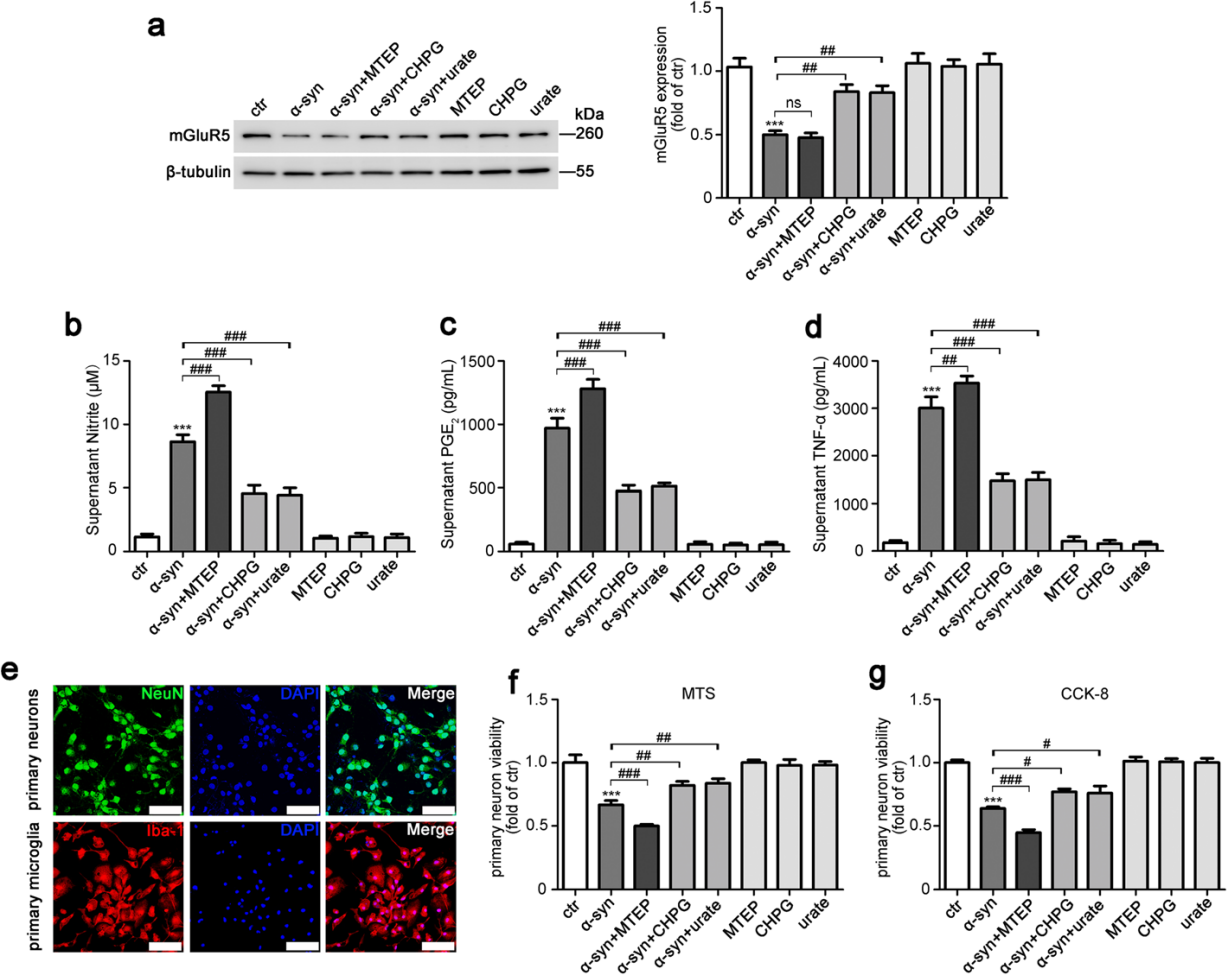
Activation of mGluR5 partially protected neurons from neurotoxicity caused by α-syn-induced microglia activation. **a**–**d** Primary microglia were infected with LV-NC or LV-α-syn followed by MTEP (100 μM), CHPG (150 μM), or urate (200 μM) treatment for 24 h, and mGluR5 protein expression was detected by western blotting (**a**, left). Quantification of mGluR5 level was normalized to β-tubulin (**a**, right). NO (**b**), PGE_2_ (**c**), and TNF-α (**d**) levels in the culture supernatants were measured by ELISA. Cells infected with LV-NC without drugs administration served as control (**a**–**d**). **e** The purity of primary neurons and primary microglia were evaluated by immunofluorescence detection of NeuN (top, green, scale bar, 50 μm) and Iba-1 (bottom, red, scale bar, 25 μm), respectively. Cell nuclei were stained with DAPI. **f**, **g** Primary neurons were incubated for 24 h with conditioned medium from cultures of rat primary microglia infected with LV-α-syn or LV-NC followed by MTEP (100 μM), CHPG (150 μM), or urate (200 μM) treatment. Cell viability was measured with the MTS assay (**f**) or CCK-8 assay (**g**). Medium from cultures of microglia infected with LV-NC served as control (**f**, **g**). Data shown in this figure represent mean ± SEM (*n* ≥ 3). The statistical significance was determined by one-way ANOVA followed by Dunnett’s test. ***p < 0.001 versus control (ctr); ^#^*p* < 0.05, ^##^*p* < 0.01, and ^###^*p* < 0.001 versus the LV-α-synuclein (α-syn) infection; ns, not significant

Microglia activation promotes α-syn pathology by increased NO production, which in turn induces nitration of α-syn in neighboring neurons and results in cell death [40]. Urate is an end product of purine metabolism in humans, and the urate level is considered a biomarker for PD risk that has the therapeutic potential [41]. We have previously reported that urate inhibits microglia activation to protect neurons in an LPS-induced model of PD [23]. To examine the effect of mGluR5 on α-syn-induced neurotoxicity from microglia activation, by which as a potential target of urate, we first collected cells and the culture medium from primary microglia infected with α-syn after urate, CHPG, or MTEP treatments, to measure the intracellular level of mGluR5 and released inflammatory factors. CHPG treatment inhibited the level of α-syn-induced mGluR5 degradation, consistent with the results shown in Fig. 3, and urate treatment exerted a similar function as CHPG by decreasing α-syn-induced mGluR5 degradation; however, treatment with MTEP had little effect on it (Fig. 5a). Activation of mGluR5 by treatment with CHPG partially suppressed α-syn-induced inflammation, including the levels of NO, PGE_2_, and TNF-α, and treatment with urate also attenuated the inflammatory response stimulated by α-syn. Blocking mGluR5 with MTEP aggravated the inflammatory effects of α-syn (Fig. 5b–d). Next, we investigated whether activation of mGluR5 protected neurons from the toxic effects of α-syn-activated microglia. The collected medium from microglia with the above treatments was used as the conditioned medium for co-cultures with primary neurons to examine neuroprotection. The purity of the primary neurons and microglia cultures was determined by immunocytochemical detection of NeuN and Iba-1, respectively (Fig. 5e). As expected (Fig. 5f, g), the conditioned medium from α-syn-overexpressed primary microglia cultures reduced neuron viability as determined by the MTS or CCK-8 assay, while the conditioned medium from either CHPG- or urate-treated primary microglia with α-syn transfection cultures increased viability. MTEP aggravated the cell viability changes caused by α-syn transfection. These results suggested that pro-inflammatory cytokines released into the culture medium by microglia upon α-syn stimulation had a negative effect on the viability of primary neurons, while activation of mGluR5 by CHPG or urate treatment partially counteracted these effects.

### Activation of mGluR5 exerted the anti-inflammatory effects in an LPS-induced rat PD model

**Fig. 6:**
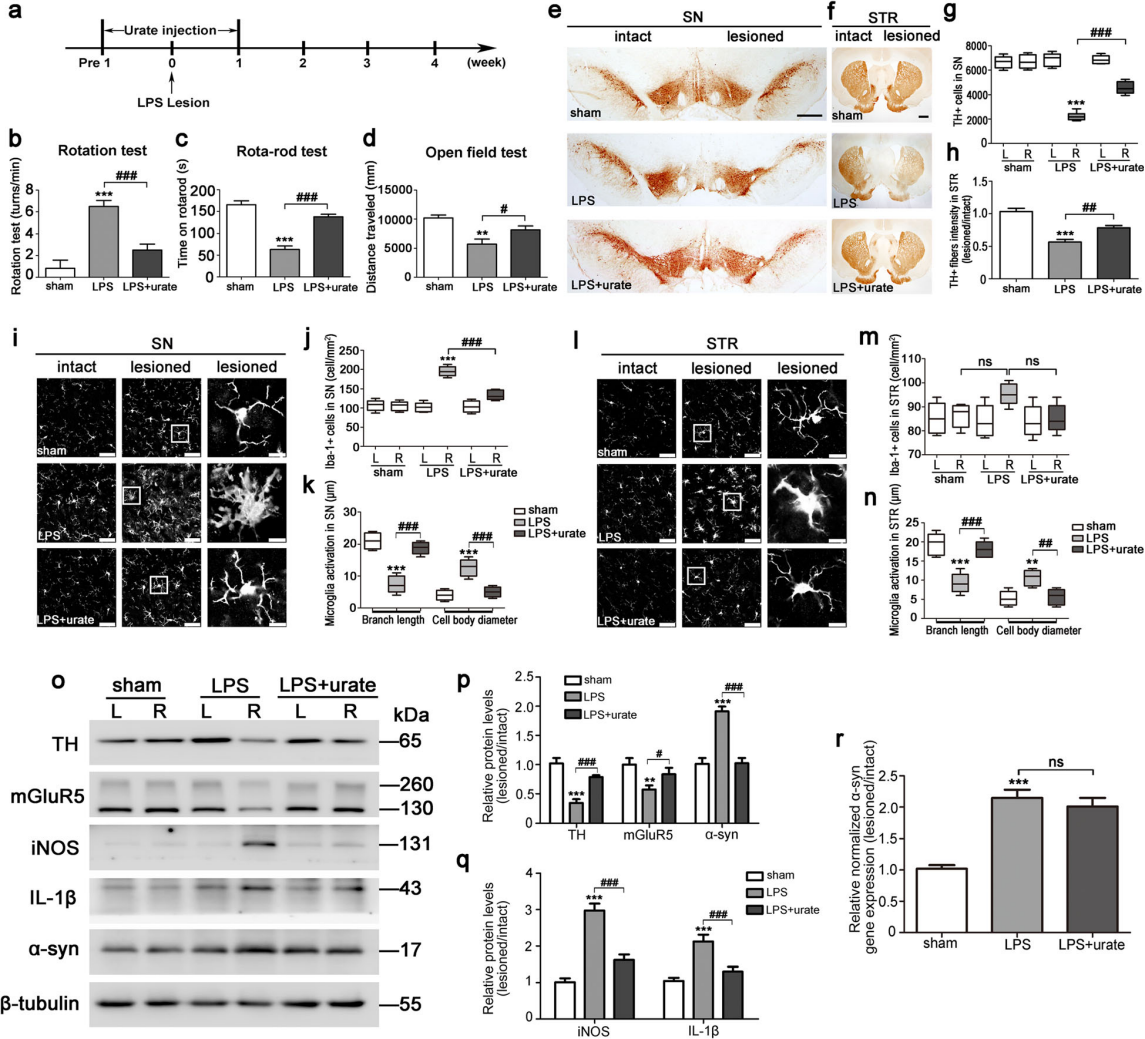
Activation of mGluR5 exerted the anti-inflammatory effects in an LPS-induced rat PD model. **a** Schematic illustration of the administration of urate to LPS-treated rats. **b**–**d** The apomorphine-induced rotational test (**b**), the rota-rod test (**c**), and the open field test (**d**) were performed 4 weeks later. **e**–**h** Representative images of TH immunoreactivity in the SN (**e**, scale bar, 500 μm) and STR (**f**, scale bar, 1000 μm). Quantification of TH-positive cells in SN was shown as the number of TH-positive cells in intact side and lesioned side (**g**). Quantification of TH-positive fibers in STR was shown as the ratio of the lesioned to the intact side (**h**). **i**–**n** Representative images of Iba-1 immunofluorescence staining in SN (**i**, scale bar, 50 μm) and STR (**l**, scale bar, 50 μm). Quantitative analysis of Iba-1-positive cells in SN (**j**) and STR (**m**), and the quantification of branch length and cell body diameter of Iba-1-positive cells in lesioned side of SN (**k**) and STR (**n**). **o**–**q** Western blotting analysis of brain lysates in SN were detected by various antibodies (**o**). Quantification of protein expression was normalized to β-tubulin (**p**, **q**). **r** The mRNA level of α-syn in SN region was analyzed by quantitative real-time PCR and normalized to that of GAPDH. α-syn, α-synuclein. Data shown in all panels represent mean ± SD (*n* ≥ 6). The statistical significance was determined using one-way ANOVA followed by Dunnett’s test. Rats injected with vehicle served as sham. **p < 0.01, ***p < 0.001 versus sham group; ^#^*p* < 0.05, ^##^*p* < 0.01 and ^###^*p* < 0.001 versus LPS lesioned group; ns, not significant

Based on the above in vitro findings, we further confirmed whether mGluR5 played a role in the neuroprotective effects of α-syn-induced microglia activation in vivo. LPS, which can directly activate microglia, was used to create a model of neuroinflammation-induced PD with augmentation of α-syn expression (Figs. 1g and 3). The treatment scheme was shown in Fig. 6a. Apomorphine administration produced rotation behavior 4 weeks after LPS treatment, and LPS-injected rats showed a reduced time on the rota-rod, indicating unilateral damage to the right SN and a decline in motor coordination with fatigue resistance. The open field test was also performed, and showed locomotor deficits with LPS treatment of rats. Urate administration significantly alleviated the functional deficits caused by LPS treatment (Fig. 6b–d). Immunohistochemical analysis showed that the number of TH+ neurons in the LPS-injected SN and the intensity of TH+ fibers in lesioned STR regions of urate-treated rats were increased by 98.50% (*p* < 0.0001) and 38.50% (*p* = 0.0057), respectively, compared with vehicle-treated rats 4 weeks after LPS treatment (Fig. 6e–h). Immunofluorescence analysis showed that urate treatment inhibited the number of increased Iba-1+ microglia in SN region (Fig. 6j), not in STR region (Fig. 6m). Also, urate reversed morphological changes from the ramified form to the reactive form in both SN (Fig. 6i–k) and STR regions (Fig. 6l–n) of the LPS-injected side, consistent with our previous report [23]. Following these experiments, western blotting analysis showed that LPS increased the protein levels of iNOS (1.95-fold increase, *p* < 0.0001) and IL-1β (1.03-fold increase, *p* = 0.0008), and decreased mGluR5 expression (0.43-fold decrease, *p* = 0.0049) with enhanced α-syn expression (0.89-fold increase, *p* = 0.0003) in vivo, accompanied by the reduction of TH (0.55-fold decrease, *p* = 0.0009), which were reversed by urate treatment (Fig. 6o–q). Additionally, we detected that the mRNA level of α-syn in SN samples with LPS injection showed a 1.1-fold increase (*p* = 0.0002) as compared to the sham group, but the treatment with urate did not affect it (*p* = 0.2796) (Fig. 6r). Taken together, the results indicated that the interrupted anti-inflammatory effect of mGluR5 contributed to neuroinflammation involving a complex with α-syn in an LPS-induced rat PD model.

### Activation of mGluR5 exerted the anti-inflammatory effects in an AAV-α-syn-induced rat PD model

Overexpression of α-syn using viral vectors represents an alternative approach to model PD-like pathology in rats to study neuron-released α-syn engulfed by microglia [42]. To directly determine the functional consequence of mGluR5 in α-syn-induced microglia activation in vivo, we detected the inflammatory response in the progression of the PD model. AAV 9 virus encoding the α-syn gene (AAV-α-syn) or GFP (AAV-GFP) was injected into the right side of SN, then the behavioral performance of rats was assessed and the animals were then euthanized for pathophysiological analysis at different time points (Additional file 3: Figure S2a). Compared with the GFP-infected group, animals in the α-syn overexpression group showed progressive functional deficits in the amphetamine-induced rotation test, rota-rod test, and open field test, which were significant from 8 weeks and maintained at 16 weeks (Additional file 3: Figure S2b-d). The reduced number of TH+ neurons in the SN and the density of TH+ fibers in the STR detected by immunohistochemical analysis revealed a progressive loss of nigral DA neurons after α-syn overexpression (Additional file 3: Figure S2e-h). Dramatically, the inflammatory response was correlated with progressive degeneration. AAV-α-syn injection caused the higher level of α-syn protein and the reduced expression of mGluR5, accompanied by an alteration in TH expression. Meanwhile, the expression levels of iNOS, IL-1β, and TNF-α were increased at 10 days after injection, and reached a peak at 4 weeks in the microglia population, while at 8 weeks and the following times, this effect vanished (Additional file 3: Figure S2i-k). This evidence indicated that the pathological process of PD was accompanied by a highly localized inflammatory response in reactive microglia at an early time point, consistent with the previous report [43].

**Fig. 7:**
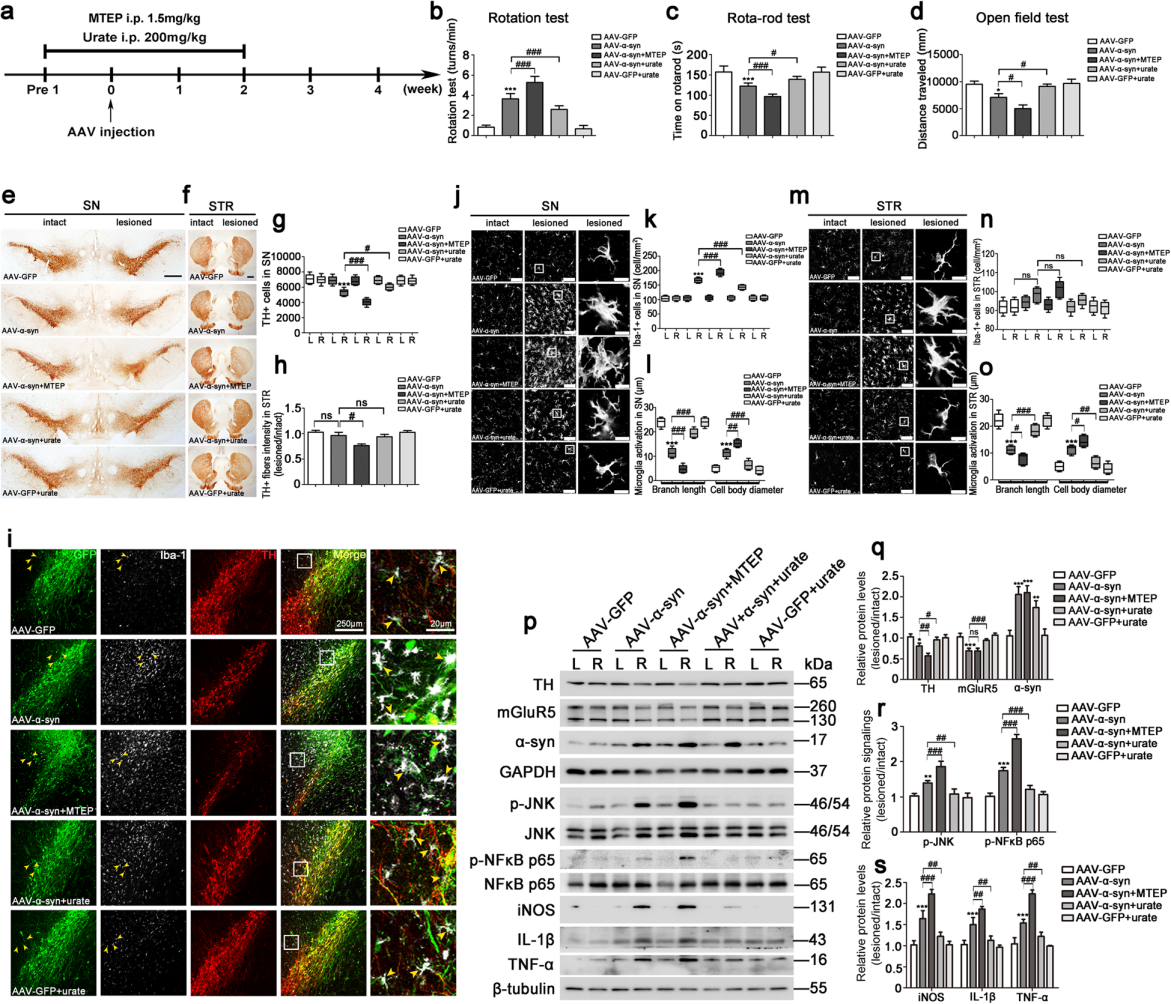
Activation of mGluR5 exerted the anti-inflammatory effects in an AAV-α-syn-induced rat PD model. **a** Schematic illustration of the administration of MTEP or urate to AAV-α-syn-injected rats. **b**–**d** The apomorphine-induced rotation test (**b**), the rota-rod test (**c**), and the open field test (**d**) were performed at 4 weeks after AAV injection. **e**–**h** Representative images of TH immunoreactivity in the SN (**e**, scale bar, 500 μm) and STR (**f**, scale bar, 1000 μm). Quantification of TH-positive cells in SN was shown as the number of TH-positive cells in intact side and lesioned side (**g**). Quantification of TH-positive fibers in STR was shown as the ratio of the lesioned to the intact side (**h**). **i** α-syn-GFP (green), Iba-1 (gray), and TH (red) were immunolabeled in SN at 4 weeks after AAV-GFP or AAV-α-syn injection accompanied by MTEP or urate administration, and images were captured by confocal microscopy. Arrowheads indicate intracellular α-syn in microglia. Scale bar, 250 μm or 20 μm. **j**–**o** Representative images of Iba-1 immunofluorescence staining in SN (**j**, scale bar, 50 μm) and STR (**m**, scale bar, 50 μm). Quantitative analysis of Iba-1-positive cells in SN (**k**) and STR (**n**), and the quantification of branch length and cell body diameter of Iba-1-positive cells in lesioned of SN (**l**) and STR (**o**). **p**–**s** Proteins from tissue lysates were analyzed by western blotting (**p**). Quantification of protein expression was normalized to GAPDH (TH, mGluR5, α-syn) and β-tubulin (iNOS, IL-1β, TNF-α). The p-JNK and p-NF-κB p65 levels were normalized to total JNK and NF-κB p65, respectively (**q**-**s**). Data shown in all panels in this figure represent mean ± SD (*n* ≥ 6). The statistical significance was determined using one-way ANOVA followed by Dunnett’s test. Vehicle groups with AAV-GFP virus delivery served as control. *p < 0.05, ** < 0.01, and ***p < 0.001 versus AAV-GFP delivery; ^#^*p* < 0.05, ^##^*p* < 0.01, and ^###^*p* < 0.001 versus the AAV-α-synuclein (α-syn) delivery; ns, not significant

To further characterize the role of inflammation intervention in the early stage of PD, we chose the peak of inflammation at 4 weeks to interfere with MTEP or urate treatment (Fig. 7a). The functional deficits in the amphetamine-induced rotation test, rota-rod test, or open field test were detected after α-syn overexpression (Fig. 7b–d), and immunohistochemical analysis showed that the number of TH+ neurons and the density of TH+ fibers in the AAV-α-syn-injected side of the rats were decreased by 22.35% (*p* = 0.0003) as compared to vehicle-treated rats. However, there was no obvious density of TH+ fibers changes in the AAV-α-syn-injected side compared with the intact side (*p* = 0.4818) in STR. Moreover, blockade of mGluR5 activity with MTEP treatment exacerbated this decrease and urate alleviated these effects (Fig. 7e–h). Moreover, as shown in Fig. 7i, we detected large amounts of AAV 9-α-syn infected TH+ neurons in the SN. Interestingly, we also detected a certain amount of Iba-1+ microglia with intracellular localization of overexpressed α-syn. Correspondingly, the number of Iba-1+ microglia in the AAV-α-syn-injected side together with urate-treated rats in the SN was decreased as compared to the group with AAV-α-syn injection, and MTEP treatment exacerbated this effect (Fig. 7j, k); however, there was no evidence that the Iba-1+ microglia number was altered in STR under the indicated conditions (Fig. 7m, n). The change of microglia morphology was observed using the microglia marker Iba-1 in both the SN and STR regions. Compared with the AAV-α-syn-injected side in rats treated with AAV-α-syn only, urate treatment reversed the morphological change from the ramified form to the reactive form, but MTEP treatment further aggravated this response (Fig. 7j, l, m, o). In addition, western blotting analysis showed that α-syn increased the inflammatory responses in the AAV-α-syn-injected side of the SN as measured by increased p-JNK, p-NF-κB p65, iNOS, IL-1β, and TNF-α. Meanwhile, mGluR5 expression exhibited a 0.32-fold decrease (*p* = 0.0009) after 4 weeks with AAV-α-syn injection, and consequently, the upregulation of TH expression by urate and its downregulation by MTEP were also observed (Fig. 7p–s). Taken together, these data suggested that activation of mGluR5 played an essential role in the anti-inflammatory response to mediate neuroprotection in an AAV-α-syn-induced PD model.

### mGluR5 exerted the anti-inflammatory effects via the modulated mGluR5-α-syn interaction in vivo

**Fig. 8:**
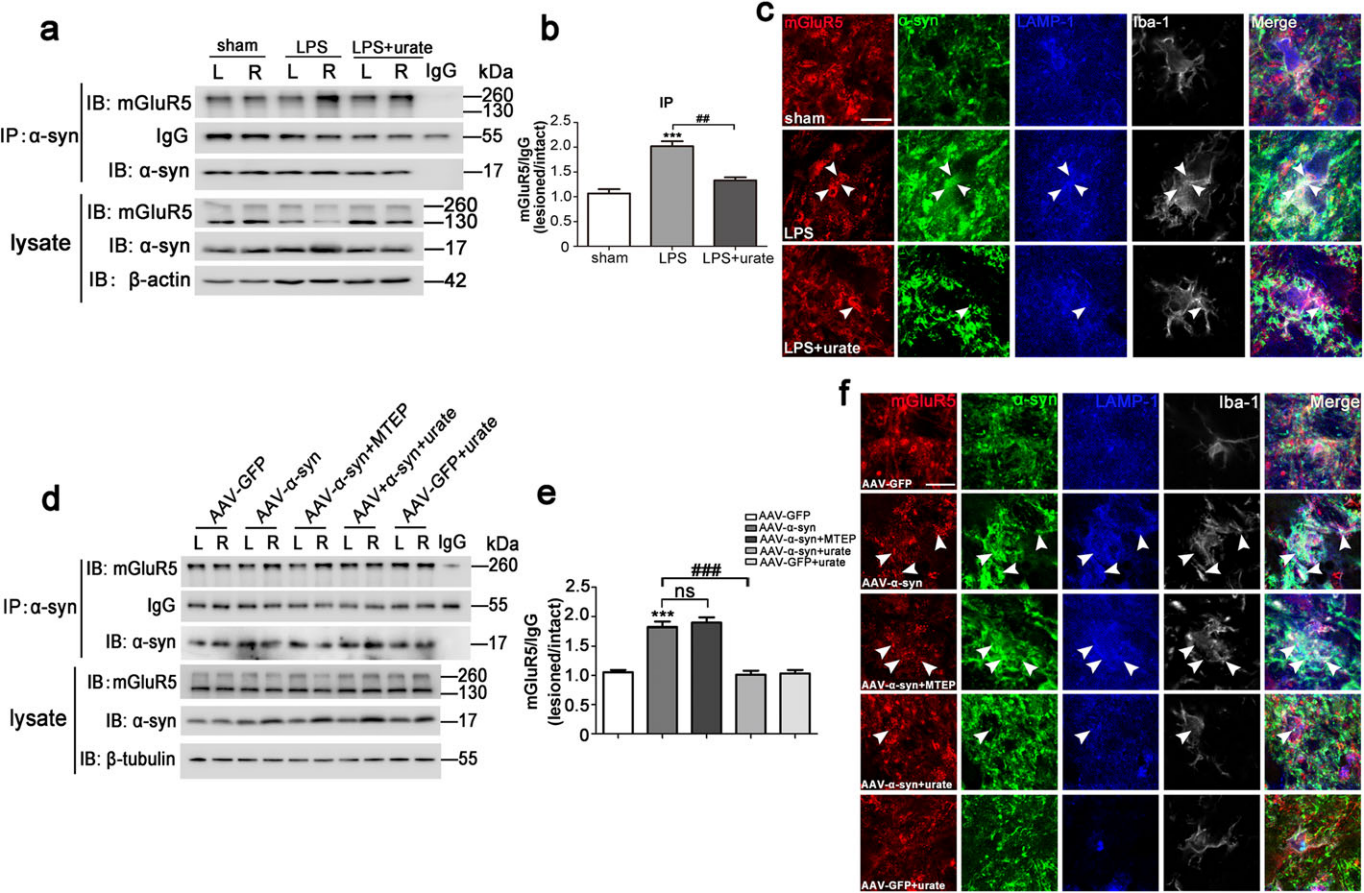
mGluR5 exerted the anti-inflammatory effects via the modulated mGluR5-α-syn interaction in vivo. **a**, **b** Brain lysates from an LPS-induced rat PD model were immunoprecipitated with control mouse IgG or anti-α-syn, and the coprecipitated proteins were analyzed by immunoblotting with anti-mGluR5 (**a**). Quantification of immunoprecipited mGluR5 level was normalized to IgG and represented as the fold different ratio (lesioned/intact) of the sham group (**b**). **c** Immunofluorescent co-stainings with mGluR5 (red), α-syn (green), LAMP-1 (blue), and Iba-1 (gray) in the lesioned SN sections. Arrowheads show examples of co-localization. Scale bar, 10 μm. Rats injected with vehicle served as sham. ***p < 0.001 versus sham group; ^##^*p* < 0.01 versus LPS lesioned group. **d**, **e** Brain lysates from an AAV-α-syn-induced rat PD model were immunoprecipitated with control mouse IgG or anti-α-syn, and the coprecipitated proteins were analyzed by immunoblotting with anti-mGluR5 (**d**). Quantification of immunoprecipited mGluR5 level was normalized to IgG and represented as the fold different ratio (lesioned/intact) of the AAV-GFP group (**e**). **f** Immunofluorescence of mGluR5 (red), α-syn (green), LAMP-1 (blue), and Iba-1 (gray) in the lesioned of SN showing the protein expression and co-localization from indicated treatments. Arrowheads indicate examples of co-localization. Scale bar, 10 μm. Vehicle groups with AAV-GFP virus delivery served as control. ***p < 0.001 versus AAV-GFP delivery; ^###^*p* < 0.001 versus the AAV-α-synuclein (α-syn) delivery; ns, not significant. Data shown in all panels in this figure represent mean ± SD (*n* ≥ 6). The statistical significance was determined using one-way ANOVA followed by Dunnett’s test

To further determine whether mGluR5 regulated microglia activation induced by α-syn via the interaction of these two proteins, in the LPS-induced rat PD model, we found that the interaction between mGluR5 and α-syn was enhanced in LPS-injected SN samples, while the binding was weakened by urate administration (Fig. 8a, b). Immunofluorescent staining verified the increased co-localization of mGluR5 and α-syn in LAMP-1 positive lysosomes in microglial cells with LPS injection, which was decreased by urate treatment (Fig. 8c). Moreover, we performed co-immunoprecipitation assays using the AAV-α-syn-induced PD model. The results showed that the AAV-α-syn lesion increased the binding to mGluR5 in the SN, which was alleviated when urate was administered. The binding between mGluR5 and α-syn had little effect with MTEP treatment (Fig. 8d, e), consistent with the in vitro results shown above (Fig. 1e). Immunofluorescent staining further confirmed the increased co-localization of mGluR5 and α-syn in LAMP-1 positive lysosomes in microglial cells in the SN with AAV-α-syn injection group, which was decreased by urate treatment. However, MTEP treatment had little effect on it (Fig. 8f). Taken together, the findings showed that the association of α-syn with mGluR5 was attributed to α-syn-induced microglia activation via modulation of mGluR5 degradation in vivo.

## Discussion

Based on epidemiological, neurogenetic, and postmortem studies, a contribution of neuroinflammatory processes in the progression of PD has been proposed [44]. Extracellular α-syn secreted from neuronal cells stimulates abnormal microglia activation in many models of PD, accompanied by a large number of inflammatory factors secreted in vitro [3, 9, 10]. The interaction between microglia activation and α-syn is therefore very important in the pathogenesis of PD. Meanwhile, mGluR5 can modulate neuroinflammation to develop neuroprotective effects in microglia by reducing oxidative stress and inhibiting inflammatory cytokine release during neurodegenerative disorders [17, 45]. Thus, regulating mGluR5 might be essential in the α-syn-induced pathogenesis of the neuroinflammation and neurotoxicity in PD. In the current study, we provided evidence that activation of mGluR5 with CHPG dissociates its interaction with α-syn, leading to the increased expression of mGluR5 via reduced lysosome-dependent degradation, further downregulating α-syn-induced inflammatory signaling, and subsequently inhibiting microglia inflammation to prevent neurotoxicity. In contrast, blockade of mGluR5 with MTEP enhanced the α-syn-induced microglia inflammation by aggravated inflammatory signaling pathways, leading to an increase of the vulnerability of neurons to degeneration.

It has been reported that overexpressed α-syn-mediated neurotoxic effects contribute to the PD pathogenesis [46], and α-syn secreted by neurons has been suggested to be closely related with abnormal microglia activation in human PD brains [47]. However, the molecular mechanisms involved in α-syn exerted neuroinflammation and neurotoxicity remain unclear. Considering that overexpressed α-syn is one of the major pathological hallmarks in PD, in the present study, we used a method of microglia activation by overexpressing α-syn. We confirmed that α-syn activated both BV2 microglial cells and primary microglia, including causing morphological changes and enhanced production of pro-inflammatory cytokines such as NO, PGE_2_, and TNF-α, leading to neuronal cell death. We further found that CHPG treatment partially inhibited α-syn-induced inflammatory response in both BV2 microglial cells and primary microglia, and subsequently protected neurons against cytotoxicity induced by microglia activation, suggesting that regulating mGluR5 is essential for α-syn-induced pathogenesis of the neuroinflammation. Indeed, we found that α-syn interacted with mGluR5 in microglia, and the interaction of these two proteins was partially dissociated by CHPG or urate treatment for mGluR5-mediated anti-inflammation (Figs. 1e and 8). Furthermore, the key point residues on the intracellular C-terminal domain of mGluR5 and the C-terminal region of α-syn for protein-protein docking were predicted for this interaction. However, these potential interacting residues need to be experimentally validated in future studies. All these results indicated that α-syn interaction with mGluR5 resulted in the dysfunction of mGluR5-mediated anti-inflammation and consequential increase in α-syn-induced microglia activation, suggesting that inhibiting microglia activation by targeting the interaction between α-syn and mGluR5 may be an effective therapeutic strategy for neuroprotection. Certainly, α-syn deficiency by short-hairpin RNA (shRNA) targeting endogenous α-syn is required for confirming the effect of mGluR5 on α-syn-induced neuroinflammation in further studies.

Previous studies showed that alterations in the levels of mGluR5 in selected brain regions cause increased α-syn accumulation in patients with DLB or PD and α-syn transgenic mice [18]. The vulnerability of hippocampal neurons to α-syn and Aβ might be mediated by mGluR5 [19]. These studies suggest that the interaction of α-syn with mGluR5 mediates receptor expression. Here, we found that LPS treatment increased α-syn expression and further increased the mGluR5-α-syn interaction to decrease receptor expression. Although it has been reported that α-syn can be degraded by both proteasomes and lysosomes, the latter have emerged as the most relevant degradative pathway in PD pathomechanisms [48]. Indeed, it has been shown that the clearance of α-syn, which was enhanced under conditions of increased protein burden, was mediated by the endosome-lysosome pathway or autophagy-lysosome pathway for α-syn degradation [49]. In the present study, we further found that overexpression of α-syn in microglia caused the degradation of mGluR5 by lysosomes, rather than by proteasomes, as shown by blockage of mGluR5 degradation by the lysosomal inhibitor, NH_4_Cl, but not by the proteasome inhibitor, MG132 (Fig. 4c, d). The co-localization of mGluR5 with α-syn was detected in lysosomes as merged with LAMP-1, a marker of lysosomes (Fig. 4f). These results suggest that the association between mGluR5 and α-syn results in lysosomal targeting of mGluR5 for degradation to aggravate the α-syn-induced microglia inflammatory response. It was reported that prolonged exposure of the ligand leads to “desensitization” of most GPCRs such as mGluR5, and subsequent endocytosis is crucial for either the “resensitization” or “downregulation” of that receptor via lysosomal degradation [50, 51]. In our study, it might be speculated that α-syn could drive mGluR5 degradation via the endosome-lysosome pathway, which should be the objective of future studies. We showed the increased expression of α-syn at both the mRNA and protein levels in an LPS-induced rat model of PD, further suggesting that α-syn maintains a high level in spite of targeting lysosomal degradation. This might be due to that the increased mRNA level of α-syn in combination with α-syn engulfed by microglia [52] was greater than the protein level of α-syn taken to the lysosome for degradation. The inflammatory response induced by α-syn accounted for the impaired function of mGluR5 by lysosomes, indicating that activation of mGluR5 plays an essential role in counteracting α-syn-induced neuroinflammation. Additionally, the urate level is considered as a biomarker for PD risk and therefore has the therapeutic potential [41]. Exogenously administered urate penetrated the compromised blood-brain barrier [53]; also, inflammation induced by LPS can disrupt the blood-brain barrier [54]. It might be speculated that, urate could penetrate the blood-brain barrier disrupted by LPS treatment to target the central nervous system for neuroprotection associated with microglia inflammation. Our previous study showed that intracellular accumulation of urate uptaken by urate transporters such as Glut9 and URAT1 was required for the anti-inflammatory effect in microglia [23]. It is likely that urate could be transported to microglia to regulate mGluR5-α-syn interaction via binding with drug-like potencies to amino acid residue hot spots [55] such as Arg and Lys on mGluR5, and Glu and Asp on α-syn (Additional file 2: Table S), or post-translational modification for mGluR5 and α-syn such as phosphorylation [56–59], which resulted in an increased expression of mGluR5 for anti-inflammation. The details that how urate could intervene on the specific mechanism of this interaction deserve further investigation. Taken together, this study indicated that urate reduces lysosome-dependent mGluR5 degradation by dissociation from α-syn (Fig. 8), which provides a novel mechanism of urate-regulated anti-inflammation, and also suggests that urate might represent an effective therapeutic strategy by targeting these two protein interactions for neuroprotection.

**Fig. 9:**
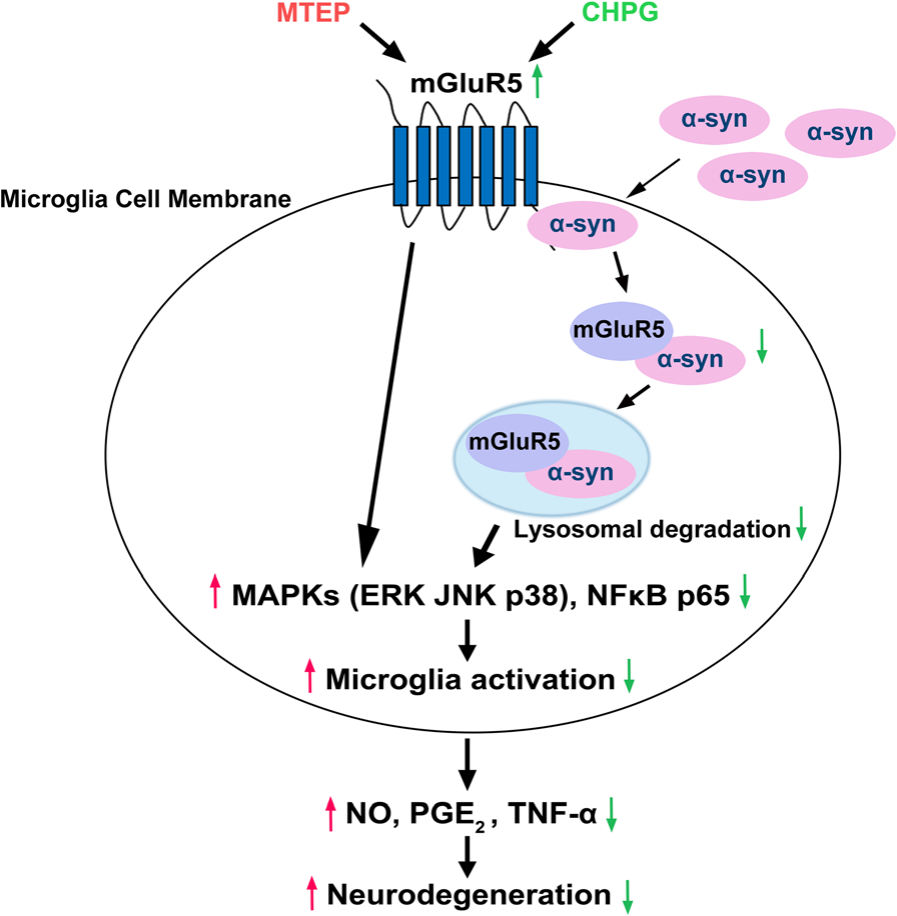
Schematic graph for the role of mGluR5 in mediating α-syn-induced microglia inflammation to protect from neurotoxicity. Activation of mGluR5 with CHPG dissociates its interaction with α-syn, leading to the increased expression of mGluR5 via reduced lysosome-dependent degradation, further downregulating the related inflammatory signaling, and subsequently inhibiting microglia inflammation to prevent neurotoxicity. In contrast, blockade of mGluR5 with MTEP enhanced the α-syn-induced inflammation by aggravated inflammatory signaling, leading to an increase of the vulnerability of neurons to degeneration

It was previously reported that signaling factors such as NF-κB, ERK, JNK, and p38 play crucial roles in the process of α-syn-induced microglia activation [60–62]. Consequently, activation of JNK or p38 MAPK is critically important towards facilitating neuronal apoptosis in AD, PD, and amyotrophic lateral sclerosis brains [63–65]. Consistent with these reports, we showed that α-syn activated microglia, which was accompanied by increased activation of these downstream signaling proteins. We further found that activation of mGluR5 with its specific agonist, CHPG, inhibited α-syn-induced downstream inflammatory signaling pathways in vitro (Fig. 2), but further activated AKT phosphorylation, suggesting that suppressing α-syn-induced inflammation by mGluR5-mediated signaling may play a protective role in microglia survival in the process of inflammatory responses. Taken together, our study showed that activation of mGluR5 dissociates its interaction with α-syn, leading to increased expression of the receptor via reduced lysosomal degradation, further downregulating the related inflammatory signaling, and subsequently inhibiting microglia activation to prevent neurotoxicity in PD models (Fig. 9).

Interestingly, differently from CHPG and urate stimulations, MTEP administration enhanced the α-syn-induced inflammation by aggravated inflammatory signaling pathways (Figs. 5 and 7p) without affecting its protein expression and its interaction with α-syn (Figs. 1 and 8). This is possibly due to that, CHPG bound to the VFT region of mGluR5 on the membrane can cause the conformational changes of the receptor, leading to the binding sites on an intracellular C-terminal tail of mGluR5 shielded from interaction with α-syn, which may significantly decrease the binding to α-syn, resulting in an increase in cell surface expression of mGluR5 to trigger the receptor-mediated anti-inflammatory signaling. However, treatment of mGluR5 with MTEP, by binding at a site on HD distinct from CHPG [14], is unlikely to cause efficient conformational changes to affect the interaction with α-syn (Fig. 1e), rather to aggravate the inflammatory signaling pathways. These findings suggest that activation of mGluR5 with CHPG is essential for counteracting microglia inflammation for PD pathogenesis via dissociation with α-syn. Further investigations are warranted to determine the role of mGluR5 in vivo experiment with AAV-α-syn-induced rat PD models when CHPG is applied. It was reported that glutamate excitotoxicity contributes to the development of PD, and the pharmacological blockade of mGluR5 has beneficial anti-kinetic effects in animal models of PD [66, 67]. Although human clinical trials using mGluR5 negative allosteric modulators have shown some limitations, recent investigations also added several more attractive small molecules to this field [68], suggesting that the identification of more strategies that facilitate mGluR5 modulation might be potentially beneficial for PD treatment. Activation of mGluR5 significantly inhibits the inflammatory response of microglia to LPS [17], and the current study further showed that activation of mGluR5 by CHPG or urate treatment inhibited microglia inflammation by decreasing the interaction with α-syn to enhance the expression of mGluR5. Studies suggested that dopaminergic neurons are more vulnerable than others in the nigrostriatal system to inflammation-mediated neurotoxicity [69, 70]. These results provide a clue that the differential modulation of mGluR5 in both neuronal excitotoxicity by MTEP inhibition and microglia inflammation by CHPG activation depending on the pathologic situation suggests a potential therapeutic strategy for PD in future human clinical trials. Of note, in an AAV-α-syn-induced rat PD model, we observed large amounts of AAV 9-α-syn infected TH+ neurons and a certain amount of AAV 9-α-syn infected Iba-1+ microglia in the SN (Fig. 7i), suggesting that there might be two reasons for causing α-syn-induced microglia inflammation by mGluR5 degradation: (1) α-syn released from AAV 9-α-syn infected neurons could be engulfed by microglia, resulting in neuroinflammation, and (2) AAV 9-α-syn infected microglia could lead to neuroinflammation. In addition, our present findings showed that activation of mGluR5 by CHPG administration partially prevented α-syn-induced inflammation (Figs. 3 and 5), suggesting that other potential signaling pathways are likely to be involved in this process. Overexpressed α-syn can also directly bind to the kinase domain on TrkB receptors, suppressing the endocytosis and trafficking of the TrkB receptors [71]; accordingly, α-syn may also target other functional proteins for degradation in response to α-syn-induced inflammation. Toll-like receptors (TLRs) and P2X7 are expressed in microglia [72, 73]. Recently the study showed that α-syn activates microglia through TLR2 and P2X7 [74, 75], and TLR4-NF-κB signaling pathway is involved in the process of microglia activation induced by α-syn [5]. Moreover, mGluR5 has been found to be involved in LPS-induced TNF-α secretion in microglia with functional TLR4 [76]. These findings raise the possibility that mGluR5 may regulate α-syn-induced neuroinflammation dependent or independent of TLRs and P2X7, which should be further investigated. It also has been reported that induction of astrogliosis by activated microglia is associated with the downregulation of mGluR5 [77]; thus, future studies might be required to determine whether the interaction of mGluR5 with α-syn also occurs in astrogliosis to regulate neuroinflammation.

## Conclusions

In summary, we found that mGluR5 played an essential role in counteracting α-syn-induced neuroinflammation. This effect was based on the mGluR5-α-syn interaction and mGluR5 degradation by the lysosomal pathway induced by α-syn. This study provides a novel mechanism involving the dissociation of the mGluR5-α-syn complex in microglia by enhanced mGluR5 expression, which may be an effective therapeutic strategy for neuroprotection.

## Supplementary Information

**Additional file 1: Figure S1.** The α-syn level in the culture medium of α-syn-overexpressed HEK293T cells. HEK293T cells were transfected with myc-α-syn for 48 h, and the culture medium was collected for ELISA. Cells transfected with vector administration served as control. α-syn: α-synuclein. ^***^p < 0.001 versus control (ctr).

**Additional file 2: Table S.** The hydrogen bonds and other intermolecular interaction involved during mGluR5 and α-syn interaction.

**Additional file 3: Figure S2.** Inflammation in the progressive neurodegeneration of AAV-α-syn-induced rat PD model. **a** The treatment of rats was shown in the scheme. **b**-**d** The apomorphine-induced rotational (**b**) and rota-rod tests (**c**), as well as open field test (**d**) were performed at indicated time periods. **e**-**h** Immunolabeling of midbrain TH+ neurons in AAV-α-syn-injected rats (10 days and 4, 8, 12, 16 weeks after injection). Representative images of TH immunoreactivity in the SN (**e**, scale bar, 500 μm) and STR (**f**, scale bar, 1000 μm). Quantification of TH-positive cells in SN, were shown as the number of TH-positive cells in intact side and lesioned side (**g**). Quantification of TH-positive fibers in STR was shown as the ratio of the lesioned to the intact side (**h**). **i**-**k** Protein expression in SN was assessed in animals subjected to the indicated treatments. Tissue lysates were analyzed by western blotting (**i**). The intensity of protein bands were normalized to GAPDH (TH, mGluR5 and α-syn) and β-tubulin (iNOS, IL-1β and TNF-α), and quantified as the ratio of the lesioned side to the intact side (**j**, **k**). Data shown in all panels in this figure represent the mean ± SD (*n* ≥ 6). The statistical significance was determined using one-way ANOVA followed by Dunnett’s test. Vehicle groups with AAV-GFP virus delivery served as control in all panels. ^*^p < 0.05, ^**^p < 0.01 and ^***^p < 0.001 versus AAV-GFP delivery group; ^###^*p* < 0.001 versus the AAV-α-syn delivery group.

## Data Availability

The datasets generated during and/or analyzed during the current study are available from the corresponding author on reasonable request.

## Abbreviations

PD: Parkinson’s disease
α-syn: α-synuclein
mGluR5: Metabotropic glutamate receptor 5
LPS: Lipopolysaccharide
NO: Nitric oxide
TNF: Tumor necrosis factor
iNOS: Inducible nitric oxide synthase
COX: Cyclooxygenase
ROS: Reactive oxygen species
IL: Interleukin
PI3K/AKT: Phosphatidylinositol 3 kinase / protein kinase B
MAPKs: Mitogen-activated protein kinases
ERK: Extracellular signal-regulated protein kinase
JNK: c-Jun N-terminal kinase
LAMP: Lysosome-associated membrane protein
LV: Lentivirus vector
AAV: Adeno-associated virus
CHPG: (RS)-2-chloro-5-hydroxyphenylglycine
MTEP: 3-[(2-Methyl-1 3-thiazol-4-yl) ethynyl]-pyridine
CHX: Cycloheximide
SN: Substantia nigra
STR: Striatum
TH: Tyrosine hydroxylase
Iba: Ionized calcium binding adaptor molecule
GAPDH: Glyceraldehyde 3-phosphate dehydrogenase
