# Supplementary figures and images for: Metabotropic glutamate receptor 5 inhibits α-synuclein-induced microglia inflammation to protect from neurotoxicity in Parkinson’s disease
Source: J Neuroinflammation. 2021 Jan 18;18:23. doi: 10.1186/s12974-021-02079-1 (PMC7814625; doi:10.1186/s12974-021-02079-1)

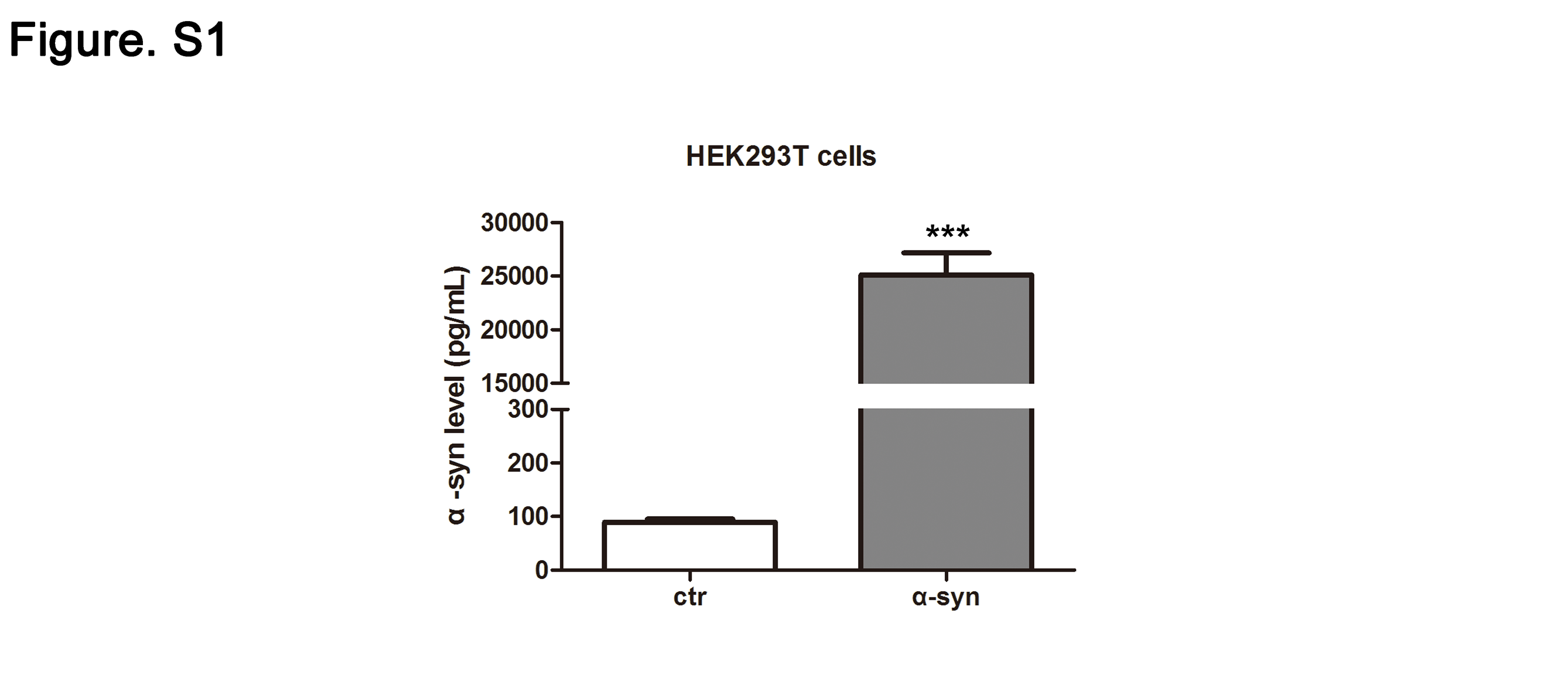

Supplement: Supplementary file 1 — Additional file 1: Figure S1. The α-syn level in the culture medium of α-syn-overexpressed HEK293T cells. HEK293T cells were transfected with myc-α-syn for 48 h, and the culture medium was collected for ELISA. Cells transfected with vector administration served as control. α-syn: α-synuclein. ***p < 0.001 versus control (ctr). [file 12974_2021_2079_MOESM1_ESM.tif]

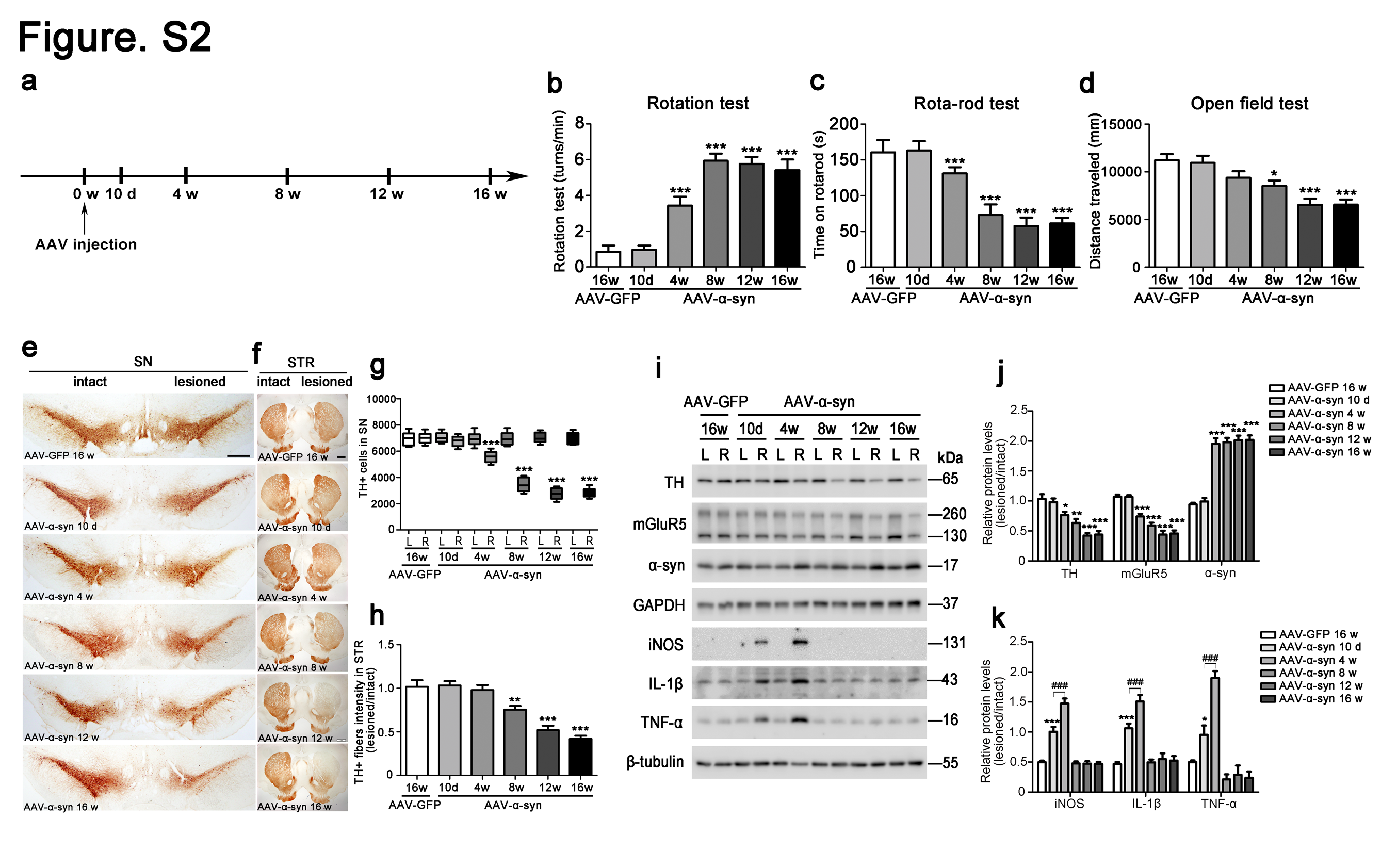

Supplement: Supplementary file 3 — Additional file 3: Figure S2. Inflammation in the progressive neurodegeneration of AAV-α-syn-induced rat PD model. a The treatment of rats was shown in the scheme. b-d The apomorphine-induced rotational (b) and rota-rod tests (c), as well as open field test (d) were performed at indicated time periods. e-h Immunolabeling of midbrain TH+ neurons in AAV-α-syn-injected rats (10 days and 4, 8, 12, 16 weeks after injection). Representative images of TH immunoreactivity in the SN (e, scale bar, 500 μm) and STR (f, scale bar, 1000 μm). Quantification of TH-positive cells in SN, were shown as the number of TH-positive cells in intact side and lesioned side (g). Quantification of TH-positive fibers in STR was shown as the ratio of the lesioned to the intact side (h). i-k Protein expression in SN was assessed in animals subjected to the indicated treatments. Tissue lysates were analyzed by western blotting (i). The intensity of protein bands were normalized to GAPDH (TH, mGluR5 and α-syn) and β-tubulin (iNOS, IL-1β and TNF-α), and quantified as the ratio of the lesioned side to the intact side (j, k). Data shown in all panels in this figure represent the mean ± SD (n ≥ 6). The statistical significance was determined using one-way ANOVA followed by Dunnett’s test. Vehicle groups with AAV-GFP virus delivery served as control in all panels. *p < 0.05, **p < 0.01 and ***p < 0.001 versus AAV-GFP delivery group; ###p < 0.001 versus the AAV-α-syn delivery group. [file 12974_2021_2079_MOESM3_ESM.tif]
