# Supplementary material for: Metabotropic glutamate receptor 5 inhibits α-synuclein-induced microglia inflammation to protect from neurotoxicity in Parkinson’s disease
Source: J Neuroinflammation. 2021 Jan 18;18:23. doi: 10.1186/s12974-021-02079-1 (PMC7814625; doi:10.1186/s12974-021-02079-1)
Supplement: Supplementary file 2 — Additional file 2: Table S. The hydrogen bonds and other intermolecular interaction involved during mGluR5 and α-syn interaction. [file 12974_2021_2079_MOESM2_ESM.pdf]

Additional file 2: Table S. The hydrogen bonds and other intermolecular interaction involved during mGluR5 and α-syn interaction.

Protein interactions (mGluR5-α-syn)			
**H-bonds**	**Pi-Pi**	**Salt-bridges**	**disulphide bonds**
R1173-NH2…OH-Y136			
R1173-NH1…OH-Y136			
I1194-O…NE2-Q134			
K1199-NZ…OE1-E131		K1199-NZ…OE1-E131	
S1196-OG…OG-S129			
E1161-OE2…OG-S129			
T1111-O…OH-Y125			
Y1200-OH…O-Y125			
D1201-OD2…O-E126			
S873-OG…OE1-E126			
R871-NH2…OE2-E126		R871-NH2…OE2-E126	
R860-NH2…OD2-D115	None	R860-NH2…OD2-D115	None
R870-O…ND2-N122			
R870-NH1…O-V118			
R870-NH1…O-D119			
R870-NH2…OD2-D119		R870-NH2…OD2-D119	
R1018-NH1…OD1-D119		R1018-NH1…OD1-D119	
R1018-NH2…OD1-D119		R1018-NH2…OD1-D119	
A985-O…OE2-E123			
C984-O…OE2-E123			
C984-O…OD2-D121			
G980-O…N-D121			
G980-N…O-D121			
